# Genome mining of WOX-ARF gene linkage in *Machilus pauhoi* underpinned cambial activity associated with IAA induction

**DOI:** 10.3389/fpls.2024.1364086

**Published:** 2024-07-24

**Authors:** Mingli Shen, Kai Zhao, Xianmei Luo, Lingling Guo, Zhirui Ma, Lei Wen, Siqing Lin, Yingxuan Lin, Hongyan Sun, Sagheer Ahmad

**Affiliations:** ^1^ College of Life Sciences, Fujian Normal University, Fuzhou, China; ^2^ Fujian Provincial Key Laboratory for Plant Eco-physiology, State Key Laboratory for Subtropical Mountain Ecology of the Ministry of Science and Technology and Fujian Province, College of Geographical Sciences, Fujian Normal University, Fuzhou, China; ^3^ College of Landscape Architecture and Art, Fujian Agriculture and Forestry University, Fuzhou, China

**Keywords:** Lauraceae, WOX-ARF linkage, miRNA binding sites, IAA response, development

## Abstract

As an upright tree with multifunctional economic application, *Machilus pauhoi* is an excellent choice in modern forestry from Lauraceae. The growth characteristics is of great significance for its molecular breeding and improvement. However, there still lack the information of WUSCHEL-related homeobox (*WOX*) and Auxin response factor (*ARF*) gene family, which were reported as specific transcription factors in plant growth as well as auxin signaling. Here, a total of sixteen *MpWOX* and twenty-one *MpARF* genes were identified from the genome of *M. pauhoi*. Though member of WOX conserved in the Lauraceae, *MpWOX* and *MpARF* genes were unevenly distributed on 12 chromosomes as a result of region duplication. These genes presented 45 and 142 miRNA editing sites, respectively, reflecting a potential post-transcriptional restrain. Overall, *MpWOX4*, *MpWOX13a*, *MpWOX13b*, *MpARF6b, MpARF6c*, and *MpARF19a* were highly co-expressed in the vascular cambium, forming a working mode as WOX-ARF complex. *MpWOXs* contains typical AuxRR-core and TGA-element cis-acting regulatory elements in this auxin signaling linkage. In addition, under IAA and NPA treatments, *MpARF2a* and *MpWOX1a* was highly sensitive to IAA response, showing significant changes after 6 hours of treatment. And *MpWOX1a* was significantly inhibited by NPA treatment. Through all these solid analysis, our findings provide a genetic foundation to growth mechanism analysis and further molecular designing breeding in *Machilus pauhoi*.

## Introduction

Plants produce shoot apical meristem (SAM) tissues during development, which contain a population of stem cells. These stem cells have the ability to produce new cells, ensuring the continuous formation of new tissues and organs as the plant develops (Bowman and Eshed, 2000; Barton, 2010). SAM is crucial for plant growth and development in the above-ground portion of the plant. The regulatory network responsible for its formation and development is highly complex, influenced by various external environmental factors and internal regulatory factors. The genes *CUC1* and *CUC2* (Aida et al., 1997), *SHOOT MERISTEMLESS* (Barton and Poethig, 1993) and WUSCHEL (WUS) (Mayer et al., 1998) have been proven to be involved in the regulatory network. The WUS gene is specifically required for maintaining the structural and functional integrity of shoot and floral meristems, which are crucial for central meristem identity (Laux et al., 1996).The WUSCHEL-related homeobox (WOX) gene family is a plant-specific class of transcription factors that belongs to a subclass of the homeobox (HOX) superfamily (Alvarez et al., 2018). Based on their phylogenetic relationships, plant WOX proteins can be classified into three categories: the WUS clade, the intermediate clade and the ancient clade (van der Graaff et al., 2009). The ancient clade includes conserved WOX genes from algae to angiosperms. The intermediate clade consists of members from ferns to angiosperms, and the modern clade is exclusive to seed plants. This classification reflects the evolutionary time when WOX genes appeared in plants (Lian et al., 2014).WOX genes are characterized by short chains of amino acids (60-66 residues) folded into a helix-loop-helix-turn-helix structure known as the Homeodomain (HD), which is responsible for DNA-binding (Gehring et al., 1990; Gehring et al., 1994; Gu et al., 2020). In addition to the HD structural domain, WOX proteins contain a unique WUS-box motif (TLXLFP) that is conserved in the WOX gene and is essential for WUS activity (Ikeda et al., 2009). The WUS-box is specific to the WUS clade members and functions as an activator and contains a C-terminal EAR domain that involves transcriptional repression. EAR-motif interacts with TOPLESS (TPL)/TPL-related (TPR) corepressor to repress the transcription of auxin-responsive genes (Szemenyei et al., 2008; van der Graaff et al., 2009). WUS (WUSCHEL) was the first gene in the WOX family to be identified. This gene is essential for maintaining the structural and functional integrity of stem and inflorescence meristems in *A. thaliana* (Laux et al., 1996). The WUS protein performs dual function, acting both as a transcriptional repressor and as an activator involved in the regulation of AGMOUS (AG) expression in *A. thaliana* (Ikeda et al., 2009). *AtWUS* transcription factors regulate the differentiation of apical stem cells by controlling auxin hormone signaling and response pathways through the regulation of histone acetylation (Ma et al., 2019). The WUS gene also affects the formation of tiller buds in rice. Xia et al. discovered that the loss of *OsWUS* function led to decreased tillering and increased apical dominance (Xia et al., 2020). Researchers have recently identified and analyzed the WOX gene families of several other plants. For example, 13, 18, 18, 14 and 31 WOX members are identified in *Ginkgo biloba* (Nardmann et al., 2009), *Populus* (Liu et al., 2014b), tea (Wang et al., 2019), *Pinus pinaster* (Alvarez et al., 2018) and maize (Zhang et al., 2010), respectively. WOX performs specific functions in key developmental processes, including embryonic patterning, stem cell maintenance, organogenesis, floral development, and hormone signaling (Haecker et al., 2004; Cheng et al., 2014; Costanzo et al., 2014; Dolzblasz et al., 2016). WOX4 has been demonstrated to be associated with cambium formation in *A. thaliana* (Suer et al., 2011) and *Populus* (Kucukoglu et al., 2017). WOX5 acts downstream of SHORTROOT (SHR)/SCARECROW (SCR) genes in maintaining the stem cell identity (homeostasis) of the root apical meristem (RAM) quiescent center in *A. thaliana* (Stahl et al., 2009). *PtrWUSa/b* and *PtrWOX13a/b/c* are expressed in the vascular cambium and differentiating xylem cells in poplar (Haghighat et al., 2024). *PtoWOX5a* is participating in the development of adventitious roots in poplar (Li et al., 2018b) and the *GhWOX13* gene affects the development of cotton fiber (He et al., 2019). WOX14 promotes the differentiation and lignification of vascular cells in the inflorescence stems of *A. thaliana* (Denis et al., 2017). However, there are limited studies on the regulatory functions of this gene family in forest trees, particularly in Lauraceae.

The primary form of auxin in plants is indole-3-acetic acid (IAA). It regulates the growth, division, and specific differentiation of cells, participating in the growth and development of various plant parts (Ljung, 2013). The highly conserved nuclear auxin signal transduction pathway is composed of the TIR1/AFB-Aux/IAA auxin co-receptors, the transcriptional co-repressor TOPLESS (TPL), and the AUXIN RESPONSE FACTORS (ARFs) (Galli et al., 2018). ARFs bind with specificity to TGTCTC auxin response elements (AuxRE) in promoters of these genes and function in combination with Aux/IAA (auxin/indole acetic acid) repressors, which dimerize with ARF activators in an auxin-regulated manner (Guilfoyle and Hagen, 2007). The ARF protein has a specific structure and can perform distinct functions, enabling it to participate in multiple signal transduction pathways and other regulatory processes. ARF typically contain three domains: the N-terminal B3-like DNA-binding domain (DBD), the transcriptional regulatory ARF domain of the middle region (MR), and the C-terminal dimerization domain (CTD) (Tiwari et al., 2003; Li et al., 2016). The DBD domain directly and specifically binds to the AuxRE of plant auxin-responsive genes, such as GH3 and SAUR. ARF domains can be categorized as activation domains (AD) or repression domains (RD) based on their functions. The CTD domain is capable of forming dimeric interactions between ARF-ARF or ARF-Aux/IAA to regulate the auxin response (Guilfoyle and Hagen, 2007). The stems of plants play a crucial role in providing support and transporting nutrients, and their growth and development are significantly influenced by auxin. *PoptrARF2.1, PoptrARF2.2, PoptrARF3.1, PoptrARF3.4, PoptrARF6.2, and PoptrARF6.3* were found to regulate the growth and development of phloem and xylem (Kalluri et al., 2007) and ARF7 as a molecular bridge of GA and auxin signaling pathways to regulate cambial development in poplar (Hu et al., 2022). *PoptrARF5* plays a key role in the development of secondary xylem (Johnson and Douglas, 2007). Moreover, PtoARF5 is able to drive the PtoIAA9-dependent cellular behaviors for secondary xylem differentiation in poplar (Xu et al., 2019). *EgrARF* of *Eucalyptus grandis* are also expressed in all parts, with the highest expression of *EgrARF3* and *EgrARF4* in the stem and phloem. *EgrARF5* is highly expressed in both xylem and phloem, while *EgrARF10* and *EgrARF19A* are highly expressed in the vascular cambium (Yu et al., 2014).

It has been demonstrated that *WOX11* and *12* are direct target genes for growth hormones in *de novo* organogenesis (Liu et al., 2014a). In addition, *WOX9* is predicted to be a downstream target gene of the MP/BDL (ARF5)-dependent auxin signaling pathway (Haecker et al., 2004). Moreover, *ARF5* promotes xylem production mainly through the direct activation of xylem-related genes and repression of *WOX4* (Brackmann et al., 2018). Meanwhile, intermediate branches of related *WOX* genes (IC-WOXs) and class A auxin response factors (A-ARFs) form various protein complexes to activate three distinct root types in *A. thaliana* (Zhang et al., 2023b). A study on the molecular mechanism of leaf flattening to form broad leaves in *A. thaliana*, has is found that redundant abaxial-rich *ARF* repressors can inhibit the expression of *WOX1* and *PRS* through direct DNA-binding (Guan et al., 2017). The aforementioned studies demonstrate a complex regulatory relationship between the *WOX* genes and *ARF*.

*Machilus pauhoi* is an evergreen broad-leaved tree of the genus Machilus in the family Lauraceae, which is characteristics by its strong budding ability, adaptability and versatility. With a straight stem and rapid growth, it is a fast-growing species with high economic value among broad-leaved trees (Chunhui et al., 2019). The rapid and high growth of forest trees is attributed to the vigorous top advantage of the plant. Recently, there have been numerous studies on the agronomic traits and physical characteristics of *M. pauhoi* (QuanLin et al., 2002; Quanlin et al., 2008; Leilei et al., 2016; Man et al., 2016; Pan et al., 2016; Yan et al., 2018; Yu-xing et al., 2018). However, there is no available report on the *MpWOX* and *MpARF* gene currently. Therefore, in this study, we identified and analyzed the WOX and ARF gene family in the *M. pauhoi* genome, including phylogenetic tree, gene collinearity, gene structure and expression pattern analysis under treatments with IAA and NPA. The aim of this study was to provide valuable information for further investigation of the functions of the WOX and ARF gene families and their interactions in the growth and development of *M. pauhoi*.

## Results

### Physicochemical properties of MpWOXs and MpARFs

A total of sixteen *MpWOX* and twenty-one *MpARF* genes were identified through HMM search and BLAST. The MpWOX and MpARF proteins were named according to their homology with *A. thaliana* WOX and ARF proteins. The analysis of the physicochemical properties of MpWOX and MpARF revealed significant differences among members of different gene families (**Supplementary Table 2**). The molecular weights of MpWOX ranged from 19864.29 to 37964.66 Da and the isoelectric points ranged from 5.76 to 9.19. The molecular weights of MpARF ranged from 41288.75 to 131242.66 Da, and the isoelectric points ranged from 5.5 to 8.54. The grand average of hydropathicity (GRAVY) index of all proteins were negative indicating that MpWOX and MpARF had good hydrophilicity. The aliphatic index was greater than 40, showing their thermostability. Subcellular prediction showed that MpWOXs and MpARFs were all localized in the nucleus, this provided a possible interaction and linkage common space.

### Phylogenetic tree of WOX and ARF in Lauraceae

**Figure 1:**
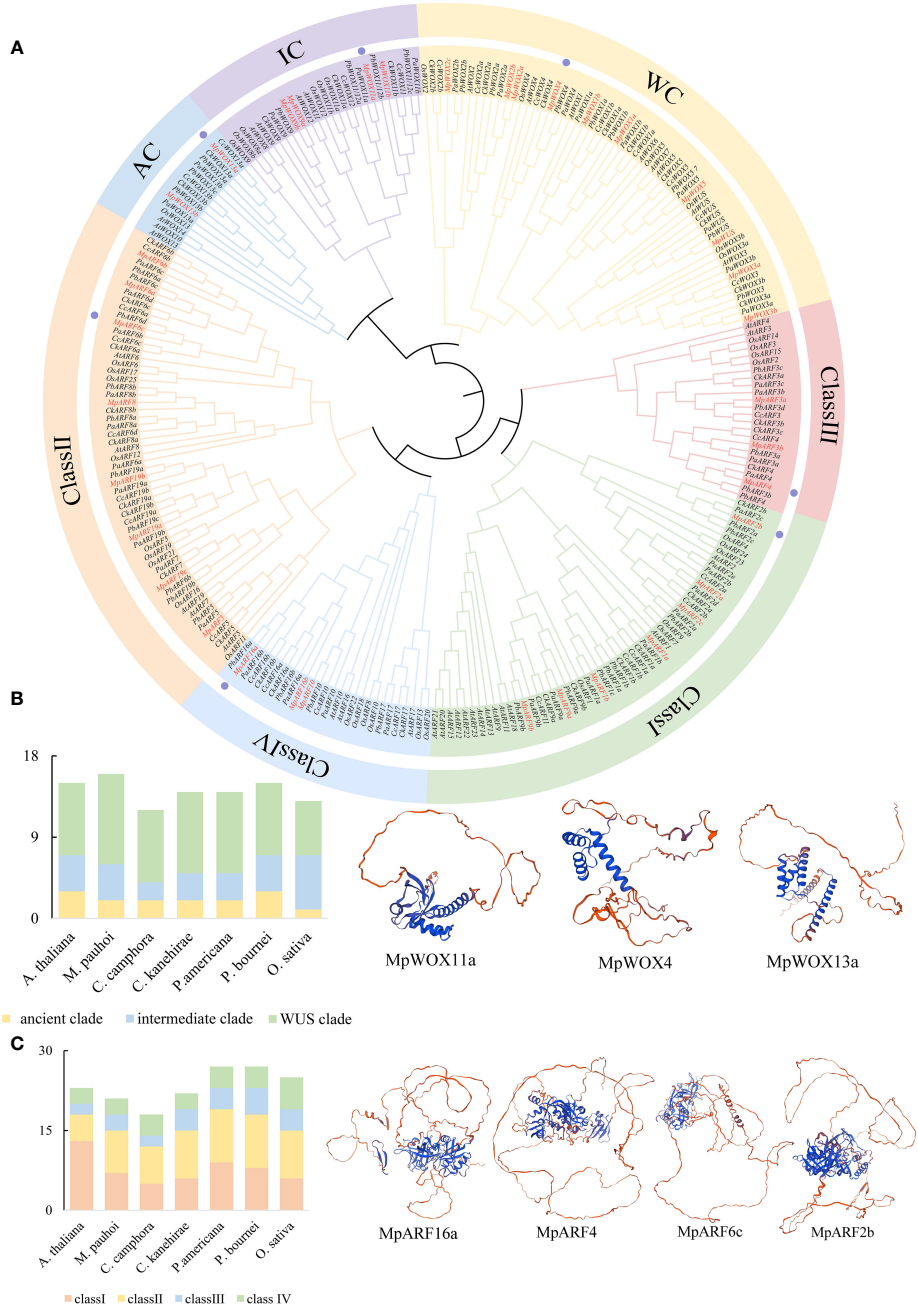
Phylogenetic analysis of WOX and ARF. **(A)** The evolutionary tree contains a total of 99 WOX and 163 ARF genes. The 99 WOX proteins can be divided into three different branches. The WC is WUS clade. IC, which stands for intermediate clade. AC is an ancient clade. There are 163 ARF proteins divided into 4 classes (I-IV). Different branches are marked with various colors. **(B)** Number of WOX gene members on different evolutionary branches in seven species. **(C)** Number of ARF gene members on different evolutionary branches in seven species. The tree contains sequences from *Machilus pauhoi* (*Mp*), *Cinnamomum kanehirae* (*Ck*), *Cinnamomum camphora* (*Cc*), *Persea americana* (*Pa*), P*hoebe bournei* (*Pb*), *Arabidopsis thaliana* (*At*) and *Oryza sativa* (*Os*). The WOX and ARF proteins in *M. pauhoi* are marked with red. In addition, the tertiary structures of some proteins are displayed, and proteins with tertiary structures are labeled with purple dots in part A.

To gain a deeper understanding of the evolutionary relationships of proteins in various species, particularly in Lauraceae, phylogenetic tree was constructed for 99 WOX and 163 ARF proteins in 7 species. The evolutionary tree was clearly divided into two main branches, WOX and ARF. The 99 WOX proteins could be divided into three branches: the WUS clade (WC), intermediate clade (IC) and ancient clade (AC). The WC contained the most WOX genes (58). The number of MpWOX proteins varied greatly in different branches (**Figure 1A**). The ancient clade contained members of the MpWOX13a and MpWOX13b. The intermediate clade contained 4 MpWOX members. The remaining MpWOXs were found in the WUS clade. According to the phylogenetic tree, 163 ARF proteins can be classified into 4 classes (I-IV). The ARF proteins in the seven species were primarily distributed in class I and class II. Class II contained the most ARF proteins (58). The *MpARF* proteins contained eight members in class II, seven members in class I, and only three members in both class III and IV (**Figure 1A**). Polyploidy in plant evolution is one of the important driving forces behind gene family expansion and the diversification of gene functions (Fernández-Mazuecos and Glover, 2017). There were numerous multi-copy genes in both WOX and ARF gene families of *M. pauhoi*, particularly *MpWOX2*, *MpARF2*, *MpARF19* and *MpARF6*, each of which has three copies in the *M. pauhoi* genome.

In addition, the number of WOX genes varied from twelve to sixteen in different species (**Figure 1B**). The number of genes in different subfamilies showed a similar trend, with the WUS clade having the highest number of genes, the intermediate clade having the next highest and the ancient clade having the lowest numbers among all species. This trend in the number of WOX genes was particularly evident in Lauraceae. The number of ARF genes varied from 18 to 27 in different species (**Figure 1C**). The number of ARF genes fluctuated greatly in Lauraceae plants. *P. americana* and *P. bournei* have the same number of ARF genes (27), while *C. camphora* contains only 18 ARF genes. Compared to *A. thaliana*, the number of class II members tended to increase in the five species of Lauraceae. In contrast, the number of class II members tended to decrease in Lauraceae, possibly due to the presence of functional redundancy in some genes.

The prediction and analysis showed that MpWOX *and* MpARF proteins of the same subfamily tended to have similar secondary structures, including alpha-helix, beta-turn, and random coil distributions (**Supplementary Table 4**; **Supplementary Figure 1**), as well as tertiary structures (**Supplementary Figure 2**). The secondary structures of proteins in different subfamilies were quite different, which was mainly reflected in the large fluctuation of alpha helix content (**Supplementary Table 4**).

### Characterizations of MpWOX and MpARF proteins

**Figure 2:**
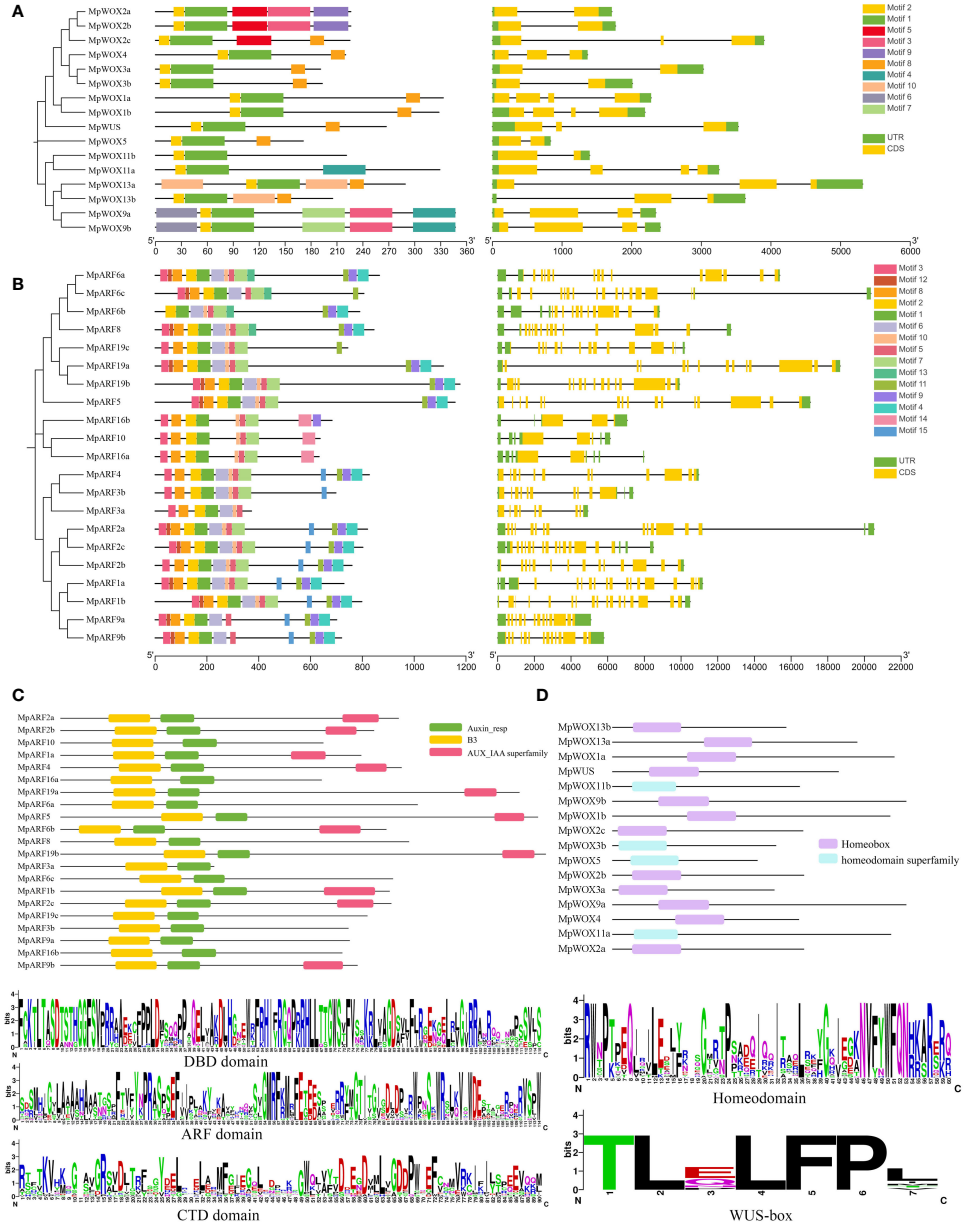
Characteristic of MpWOX and MpARF proteins. **(A, B)** denotes conserved domain and gene structure analysis of MpWOX and MpARF, respectively. **(C)** Conserved structural domains of the MpARF. Sequence logo of DBD domain, ARF domain, and CTD domain. **(D)** Conserved structural domains of the MpWOX. Sequence logo of homeodomain and the WUS-box specific to the WC member.

The conservative motif showed that MpWOX contained ten distinct motifs. Motif 2 and motif 1 were commonly found in the MpWOX proteins. Motif 10 was specifically present in the ancient clade of the MpWOX. Interestingly, motif 7 and motif 4 only existed in the intermediate clade. Motif 3, motif 8, motif 2, motif 1 and motif 5 were present in all MpARF proteins. Motif 14 was specifically present in the three MpARF proteins of class I, while all three genes of class I were missing motif 6. The results of protein structure analysis showed that members with similar phylogenetic relationships also had similar intron/exon structures. The number of introns varied between one and three in the *MpWOX* genes. The numbers of introns in the *MpARF* gene were highly variable, ranging from four (*MpARF16b*) to eighteen (*MpARF2c*). Long introns were found in both *MpWOX* and *MpARF* genes, such as *MpWOX13a*, *MpWOX2c*, *MpARF6c*, *MpARF*2a. The presence of longer introns may be due to the insertion of transposons within the genes (**Figures 2A, B**).

Analysis of the conserved protein sequences of AtWOXs and MpWOXs revealed that 31 amino acid sequences contained a conserved homeodomain with an average length of 60 aa. Further multi-sequence alignment of the amino acids in the WOX homeodomain revealed that the AtWOX and MpWOX homeodomains were highly conserved. The WUS-box (TLXLFP) is an important structure for the functioning of WC members. The WUS-box was observed in all ten WC members of *M. pauhoi* (**Figure 2D**; **Supplementary Figure 4**). The Batch CD analysis found that all MpARFs contained Auxin_resp and B3 structural domains, which further confirms the accuracy of the identified *MpARF* genes. Additionally, there were ten MpARFs that did not contain the AUX/IAA superfamily structural domain, and these genes were mainly found in class I and class II. We conducted a multiple sequence comparison among them and the results showed that all MpARFs contained DBD and ARF domains. The CTD structure of certain MpARFs were missing to varying degrees. The conserved structural domains of each gene had a relatively similar sequence composition to one another (**Figure 2C**; **Supplementary Figure 3**).

### Collinearity analysis in the current *M. pauhoi* Genome

**Figure 3:**
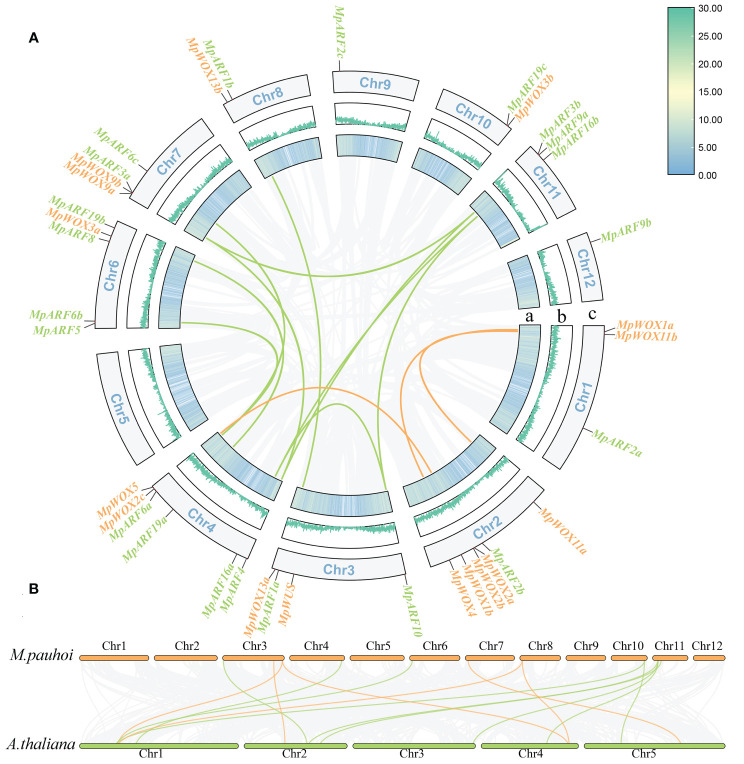
Intragenomic collinearity map of *MpWOX* and *MpARF.* **(A)** Intragenomic collinearity map of *MpWOX* and *MpARF.* a: The colored lines indicate gene density. b: The width of the blue lines represents the gene density. c: Chr1-Chr12 represent 12 chromosomes. Green and orange lines represent collinear pairs between ARF and WOX genes, respectively. Gray lines indicate all synteny blocks in the genome. **(B)** Synteny analysis of WOX and ARF genes between *M. pauhoi* and *A.thaliana*. Green and orange lines represent collinear pairs between ARF and WOX genes, respectively. Gray lines indicate all synteny blocks in the genome.

**Figure 4:**
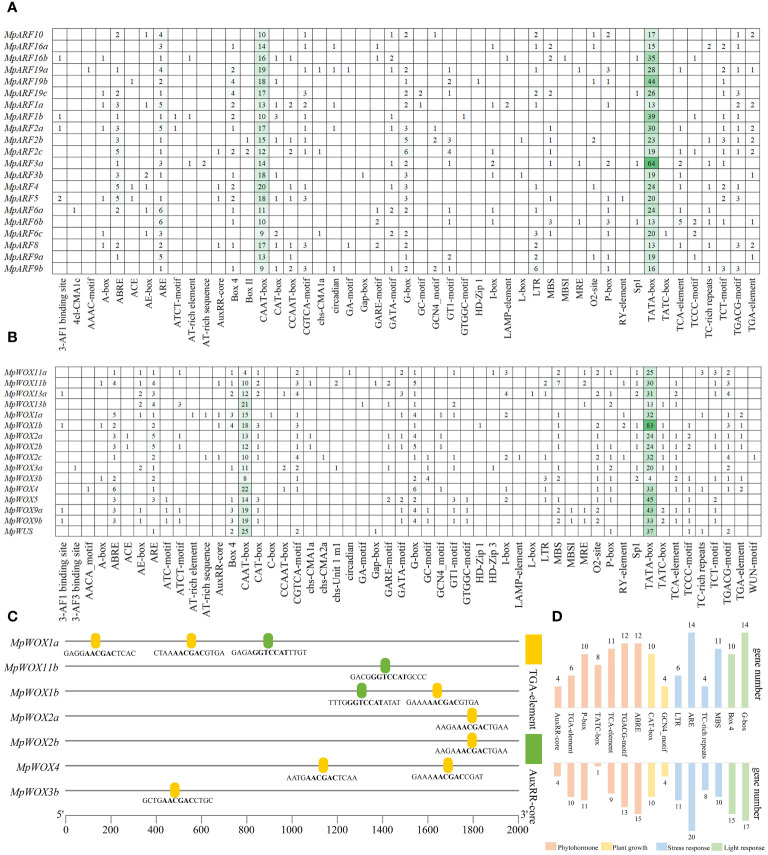
Cis-regulatory element in *MpWOXs* and *MpARFs*. **(A, B)** represent the number and type of cis-regulatory element in *MpWOX* and *MpARF*, respectively. **(C)** Structure and sequence of AuxRR-core and TGA-element in *MpWOX* promoter. **(D)** Quantitative statistics of major cis-acting elements in genes.

All *MpWOX* and *MpARF* genes were mapped to chromosomes based on the gene coordinate annotation data. The 16 *MpWOX* genes were unevenly distributed on 8 out of the 12 chromosomes and located mainly at the ends of each chromosome (**Figure 3A**). No *MpWOX* genes were located on chromosomes 5, 9, 11, and 12. Chromosome 2 boasted the highest count of *MpWOX* genes, totaling five. Collinearity analysis showed that there were three pairs of segmental duplication genes in *MpWOX*. Two of them were involved genes from WUS clades, one was involved a homologous gene from intermediate clades, and none was involved a homologous gene from ancient clades (**Supplementary Table 5**). The 21 *MpARF* genes were unevenly distributed on 11 out of the 12 chromosomes of *M. pauhoi*. Chromosome 5 was the sole chromosome that lacked any MpARF genes. Meanwhile, a total of 10 pairs of segmental duplication genes were identified among the 21 *MpARF* genes, spanning classes I-IV. These co-linear gene pairs predominantly consisted of genes that exhibit multi-copying phenomenon. Notably, chromosome 4 was involved in the most segmental duplication events, with a total of six duplication pairs of the *MpARF* gene localized on this chromosome. In addition, tandem duplication genes were not present in either the *MpWOX* genes or the *MpARF* genes. Furthermore, the collinearity between *M. pauhoi* and *A. thaliana* was analyzed (**Figure 4B**). A total of 15 pairs of collinear relationships between *A. thaliana* and *M. pauhoi*, which harbored six pairs of homologous genes for *MpWOX* and nine pairs of homologous genes for *MpARF*, respectively (**Figure 3B**).

### Cis-acting element prediction and arrangement rule in the gene promoters

1206 and 1304 cis-acting regulatory elements were predicted in the promoters of *MpWOXs* and *MpARFs*, respectively (**Figures 4A, B**). Most of these different cis-regulatory elements were involved in plant hormonal regulation, abiotic stress, plant growth, and light response. For *MpWOXs*, apart from the CAAT-box and TATA-box, the most prevalent regulatory element was the G-box, which is a universal regulatory element in plant responses to environmental stimuli and is widely present in the promoters of light-regulated genes. ABREs (regulating of abscisic acid) and AREs (anaerobic induction) were widely present in *MpWOXs*. Some auxin-responsive functional elements such as TGA-element (AACGAC) and AuxRR-core (GGTCCAT) exist in seven *MpWOX* genes (**Figure 4C**). *MpARFs* contained regulatory elements for auxin, abscisic acid, gibberellin, salicylic acid, and MeJA. In addition, it contained numerous light-responsive elements, such as G-box, Box4, and GATA-motif (**Figure 4D**). In conclusion, all the predicted results indicated that *MpWOX* and *MpARF* may play important regulatory roles in the responses or regulation by the environment, phytohormones, and abiotic stresses.

### Detection of miRNA targeting *MpWOX* and *MpARF* genes

**Figure 5:**
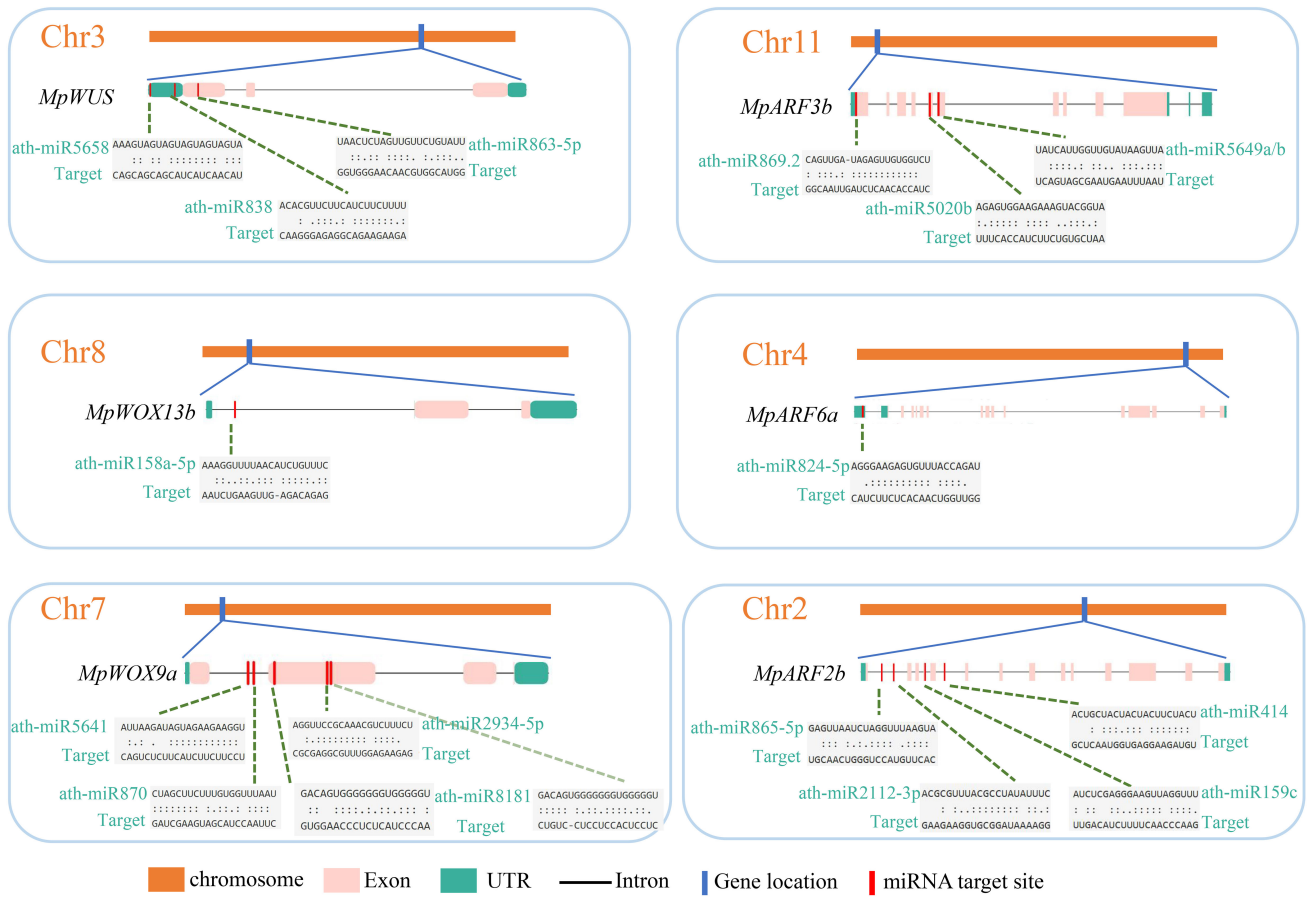
The predicted target sites of miRNA of *MpWOX* and *MpARF* gene family. Left and right represent the distribution of miRNA editing sites in *MpWOX* and *MpARF* genes, respectively. Detailed miRNA editing information for each gene can be found in **Supplementary Table 6**.

Plant miRNA is a group of 21-nucleotides that belongs to endogenous non-coding single-stranded RNA. To gain a better understanding of the role of miRNAs in the post-transcriptional regulation of *MpWOXs* and *MpARFs*, the miRNA editing sites were predicted based on the psRNATarget database. 45 and 142 miRNA editing sites were predicted in 16 *MpWOX* and 21 *MpARF* genes, respectively (**Figure 5**; **Supplementary Table 6**). Among them, there were no miRNA editing sites in *MpWOX3a*. However, there were more miRNA editing sites in *MpWUS*, *MpWOX9a*, *MpWOX9b, MpWOX11a*. *MpARF9a* contains the most miRNA-targeting sites (20). *MpARF19c* contained only one miRNA editing site. At the same time, these predicted miRNA editing sites were mainly distributed in the upstream region of the genes.

### Tissue-specificity of *MpWOX* and *MpARF* gene expression

**Figure 6:**
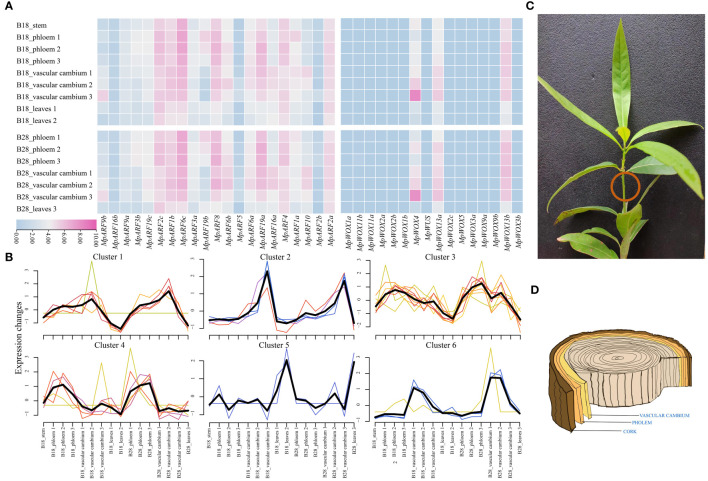
Expression patterns of *MpWOX* and *MpARF* genes in *M. pauhoi*. **(A)** The transcriptome data of *M. pauhoi* were obtained from two different lineage, B18 and B28. Among them, B18 lineage plant materials from Ganzhou, Jiangxi province, and B28 lineage plant materials from Jiande, Zhejiang province. Phloem 1: 1 year after planting; Vascular cambium 1: 1 year after planting; Leaves 1: 1 year after planting; Phloem 2: 2 years after planting; Vascular cambium 2: 2 years after planting; Leaves 2: 2 years after planting; Phloem 3: 3 years after planting; Vascular cambium 3: 3 years after planting. FPKM values are logarithmically transformed using a base of 2 for plotting the heatmap. **(B)** Gene expression trends of the *MpWOX* and *MpARF* gene. The black line represents the central trend change, and the other color lines represent the trend change of different genes. **(C)** *M. pauhoi* plants. **(D)** Schematic diagram of the phloem and vascular cambium inside the stem of *M. pauhoi*.

Based on the available transcriptome data, we analyzed the expression of *MpWOX* and *MpARF* genes in leaf, stem, phloem and vascular cambium (**Figures 6C, D**). There were significant differences in the expression of all *MpWOX* genes across different tissues. *MpWOX4*, *MpWOX1*3a and *MpWOX13b* were expressed by higher levels in all tissues compared with other members of the WOX gene family. This suggested that these three genes may act as important regulatory roles in the growth and development of these four tissues. All *MpWOX* genes exhibited similar expression profiles in two distinct lineages. In particular, the expression of *MpWOX4* gene increased sharply during the transition from vascular cambium 2 to vascular cambium 3. The change of expression level may be related to the thickening of plant growth during the later stages of growth. Compared to the *MpWOX* genes, all the *MpARF* genes, except *MpARF*5, *MpARF*2b, and *MpARF*16b, exhibited higher expression in various tissues. Among them, *MpARF*6c, *MpARF*19a, *MpARF*8, *MpARF*2c, and *MpARF*4 showed higher expression in phloem, indicating that these genes may play an important role in phloem formation. In addition, *MpARF*6b, *MpARF*6c, and *MpARF*19a were significantly expressed in the vascular cambium, suggesting that they may perform essential activities in this tissue. Similarly to *MpWOXs*, the *MpARF* genes showed similar expression patterns in different lineages (**Figure 6A**). Based on the expression trend of genes, aside from genes in cluster 4 and cluster 5, the remaining genes were mainly clustered in vascular cambium across various periods in different lineages (**Figure 6B**).

### Interaction networks of WOX-ARF with functional genes

**Figure 7:**
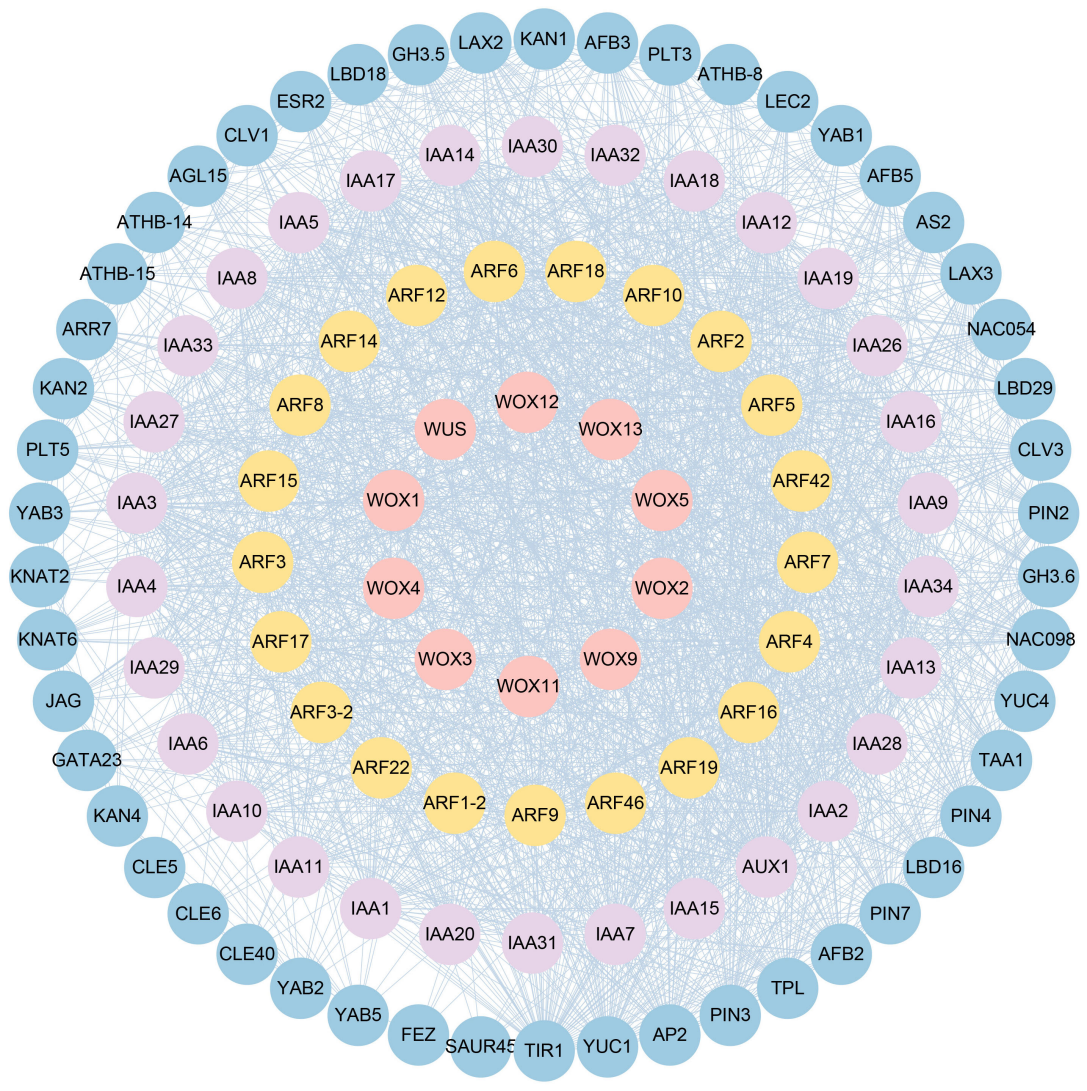
WOX-ARF protein interaction network. Interaction networks between the MpWOX and MpARF are predicted based on the homolog to *A. thaliana*.

To further understand the biological function and MpWOXs and MpARFs regulatory networks, the protein-protein interaction (PPI) of MpWOXs and MpARFs were predicted and constructed based on the interaction information of *A. thaliana* in STRING database. This interactome map consisted of the essential auxin signaling transduction components mediated by WOX-ARF proteins (**Figure 7**). TPL/TPR corepressors were first described as direct interactors of the Arabidopsis homeodomain transcription factor WUSCHEL (Kieffer et al., 2006). In auxin resting state, AUX/IAA repressors binded auxin response factors (ARFs) and repress their transcription by recruiting the TOPLESS and TOPLESS-related co-repressors (TPL/TPRs). In the presence of auxin, AUX/IAAs binded to TIR1/AFB receptors, they were quickly ubiquitinated and degraded, subsequently releasing the repression of auxin-responsive genes (Mockaitis and Estelle, 2008). Four WOX and nine ARF proteins directly interacted with TPL in the interaction network. There were also interactions between TIR1 and three WOX proteins. In conclusion, based on the predicted results of the interaction network, WOX proteins may be a potential regulatory role of ARFs in plant auxin signaling.

### *MpWOX* and *MpARF* genes response to hormone treatments

**Figure 8:**
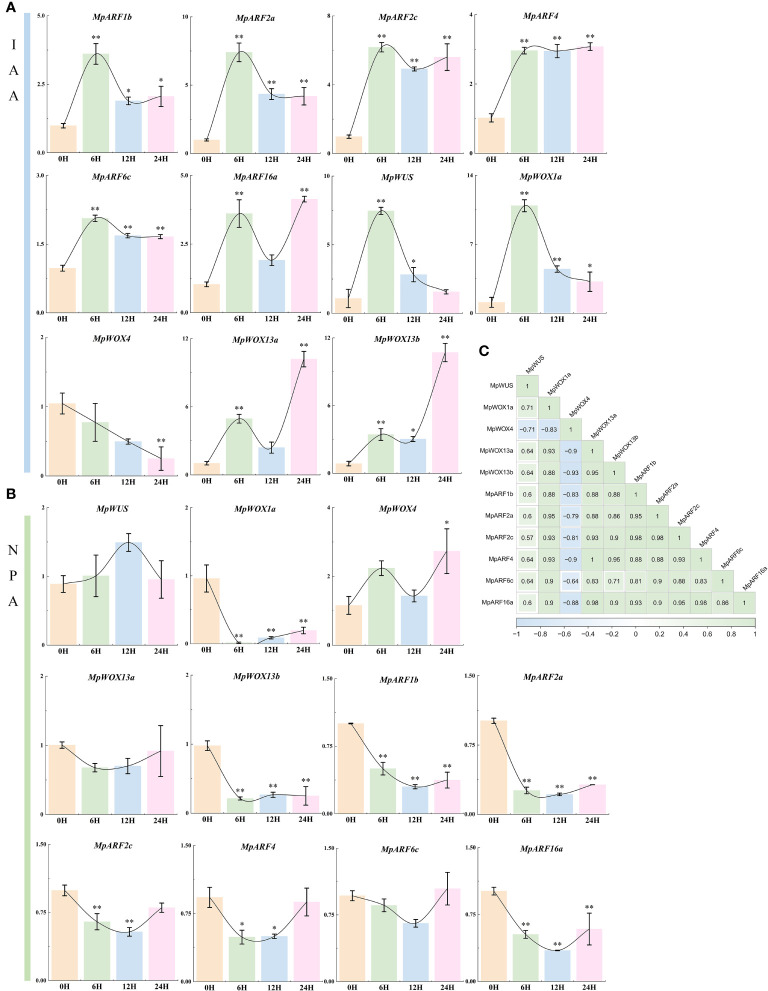
The relative expression level of *MpWOX* and *MpARF* under hormone treatment. The data is the average ± standard deviation of the repetition of the three organisms. * and * * stand for p < 0.05 and p < 0.01 respectively, by One-way ANOVAs. **(A)** The relative expression level of *MpWOX* and *MpARF* gene under IAA treatment. **(B)** The relative expression level of *MpWOX* and *MpARF* gene under NPA treatment. **(C)** Correlation Heatmap.

Based on the prediction of cis-regulatory element and PPI, there was a potential interaction between *MpWOX* and auxin. We treated 1-year-old *M. pauhoi* with exogenous hormones and analyzed their leaves using RT-qPCR. The results indicated that the expression of all *MpARF* genes, as well as *MpWOX* genes, except for *MpWOX*4, were strongly induced under IAA treatment (**Figure 8A**). The *MpARF* genes exhibited a more consistent trend in expression, with fluctuations of varying degrees following a significant increase in expression upon induction for 6 hours. The expression levels of *MpWOX13a* and *MpWOX13b* increased with the prolongation of treatment time. The expression levels of *MpWUS* and *MpWOX1a* were strongly induced after 6 hours of treatment, and showed a decreasing trend. For NPA treatment, all genes showed down-regulated expression after 6h treatment except *MpWUS* and *MpWOX4*. *MpWUS*, *MpWOX13a* and *MpARF6c* had no significant change across all time points. The expressions of *MpWOX1a*, *MpWOX13b*, *MpARF1b*, *MpARF2a*, and *MpARF16a* were inhibited at all time points (**Figure 8B**). Based on the correlation network, there were mainly positive correlations between *MpWOX* and *MpARF* genes, except for *MpWOX4* (**Figure 8C**).

## Discussion

### Variation in the numbers of WOX and ARF genes in the Lauraceae family of plants

The WOX gene is a plant-specific TF and plays a crucial role in stem cell formation, maintenance, differentiation, and organogenesis (Dolzblasz et al., 2016). ARFs play a crucial role in the auxin signaling pathway by regulating their own expression and influencing the transcriptional activity of downstream target genes. They achieve this by binding to the response elements in the promoters of auxin responsive genes (Liu et al., 2015). Although the WOX and ARF gene families have been extensively studied in some model plants, these gene families have been less studied in woody plants, especially in Lauraceae. Earlier studies have also demonstrated that ARF-WOX forms a complex in plants to regulate plant growth and development. In this study, AtWOXs and AtARFs from Arabidopsis were utilized to identify WOXs and ARFs proteins in five Lauraceae plants, revealing a range of 12 to 16 WOXs and 18 to 27 ARFs. The quantities differed less from those of *A. thaliana* [15 *AtWOX* (Haecker et al., 2004), 23 *AtARF* (Guilfoyle and Hagen, 2001)] and rice [13 *OsWOX* (Zhang et al., 2010), 25 *OsARF* (Wang et al., 2007)], suggesting that there may not have been a large-scale gene duplication event in these two gene families in Lauraceae. Members of the *MpARF* and *MpWOX* subfamilies exhibit differences in molecular weight, isoelectric point, and gene structure. These variances are commonly observed in other plants (Yang et al., 2022; Chen et al., 2023). Referring to the classification system of *A. thaliana* WOX and ARF genes, WOXs and ARFs can be categorized into different evolutionary branches, with variations in the number of genes among these branches. Deletions of *A. thaliana* homologous genes were present in both *MpWOXs* and *MpARFs* and these deletions may be attributed to their functional substitutability (Feng et al., 2021). Members of the WUS branch accounted for a large proportion of the species were counted, indicating that each member of this branch had a specific function in plant growth. At the same time, the large number of WUS clades might be attributed to gene duplication during evolution, particularly in seed plants, to ensure the normal growth of plants (Tang et al., 2017).

### Highly conserved structures of MpWOX and MpARF

The homeodomain is conserved across the WOX gene family in different species and maintains the functional integrity of the WOX gene (Kanchan and Sembi, 2020). Our results show that the HD structure is present in all MpWOX proteins. It has also been demonstrated that the typical WUS-box is limited to the WUS clade. Additionally, the WUS-box interacts with TOPLESS-type co-repressors (WUS, WOX1, WOX5) to mediate gene repression through histone deacetylation (Zhang et al., 2014; Pi et al., 2015). All members of the WUS clade have two amino acid motifs T-L at the beginning of the WUS-box, whereas non-WUS members of the WOX family show variations at this position (van der Graaff et al., 2009). In general, ARF proteins contain three domains: DNA-binding domain (DBD), middle region (MR), and C-terminal domain (CTD). All MpARFs have a typical B3 domain required for efficient binding of AuxRE and ARF domain. However, some MpARFs lack the CTD structure, which may not participate in the auxin signal transduction pathway in plants and perform independent functions (Rui-E et al., 2011). For MpARFs containing DNA-binding domain (DBD) and AUX/IAA domains, we found that these proteins were highly conserved for residues crucial for DNA and IAA binding. These highly conserved structures ensure these proteins accurately perform regulatory functions. The collinearity analysis results revealed the presence of intraspecific collinear homologous genes in both *MpWOX* and *MpARF* genes, suggesting the occurrence of gene duplication events in *M. pauhoi* during evolution, with segmental duplication being the primary mode. As a result of this intragenic segmental duplication event, the *MpWOX* and *MpARF* gene family members turn to be multi-copy.

### Cis-regulatory elements and expression patterns reveal the linkage between *MpWOX* and *MpARF*

The miRNA complements and connects with target gene mRNA to repress gene expression or cleave mRNA, thereby achieving negative regulation of the target gene. The regulatory role of miRNA on the expression of certain genes has been discovered and confirmed in *A. thaliana* (Wu et al., 2006). In our study, we find that genes with a higher number of miRNA editing sites exhibit lower expression levels in various tissues. In addition, among the *MpARF* genes, some genes lacking the complete CTD structural domains, such as *MpARF10*, *MpARF16a*, *MpARF3a*, and *MpARF19c*, had relatively low expression levels. Comparison of additional conserved structural domains revealed that these genes had a low number of motifs, and the absence of certain motifs may have impacted their normal regulatory functions. It has been demonstrated that WOX4 is located downstream of ARF7, which can directly regulate WOX4 expression and promote the activity of cambium stem cells in poplar (Hu et al., 2022). Previous studies have confirmed the existence of a complex regulatory network between WOX and ARF (Liu et al., 2014a). Some of the *MpWOX* genes contain auxin response elements, suggesting that these genes may be potentially regulated by ARF genes. In *MpWOX* genes, while canonical auxin response elements (AuxREs) are absent, they do contain AuxRR-core and TGA-elements. RhARF18 was able to directly bind the AuxRR cis-element in the *RhAG* promoter and suppress its transcription activity in *Rosa hybrida (* Chen et al., 2021). Meanwhile, ARF7 and ARF19 are able to bind to the auxin-response elements of the PHR1 promoter *in vitro* and vivo (Huang et al., 2018). WOX4 and WOX14 can regulate the division activity of cambium cells (Etchells et al., 2013). In our study, *MpWOX4* was highly expressed in the vascular cambium, which was consistent with previous research. During secondary development of *A. thaliana*, the *ARF5* is activated by auxin, regulating vascular proliferation early in development but inhibiting vascular cambium expansion later in secondary development (Li et al., 2018a). ARF5 also promotes *PIN1* expression in the pro-cambium and increases the number of vascular cambium cells (Wenzel et al., 2007; Smetana et al., 2019). *MpARF6b*, *MpARF6c*, and *MpARF19a* exhibited higher transcription levels in the vascular cambium, suggesting that these genes play crucial roles in the expansion of *M. pauhoi* cambium cells. Based on the expression trends of genes at different stages, *MpWOX* and *MpARF* genes exhibit similar expression patterns, showing high expression during various developmental stages of vascular cambium. Furthermore, the prediction results of the protein interaction network confirmed the presence of an interaction between the ARF-WOX genes. Based on the analysis results and existing research, we believe there exists a linkage-based expression regulatory pattern between WOX and ARF in *M. pauhoi*, especially playing a crucial role in regulating vascular cambium development. However, interactions between these genes require further experimentation, such as Yeast Two-Hybrid (Y2H) and Bimolecular Fluorescence Complementation (BiFC).

### The linkage of *MpWOX* and *MpARF* are binded by the joining of IAA

In the auxin signaling pathway, the Aux/IAA-TIR1-ARF signaling cascade regulates the transcription of auxin, facilitating adjustments in auxin concentration to indicate alterations in the transcriptional activity of numerous genes (Paponov et al., 2008). In addition to the Aux/IAA-TIR1-ARF signaling pathway, ARF is also involved in other signaling pathways and regulated by other transcription factors, miRNAs, and ta-siRNAs (Yan-Lin et al., 2017). Based on the prediction from the STRING database, the protein interaction network is primarily regulated by Aux/IAA, TIR1, and ARF genes. It is also suggested that the Aux/IAA-TIR1-ARF signaling pathway plays a crucial role in auxin signal regulation. In *Phoebe bournei* (Zhang et al., 2023a), the *PbWUS* gene was strongly induced by IAA treatment, which supports our conclusion. However, the expression levels of *PbWOX13a* and *PbWOX13b* decreased under IAA treatment, which differed from our results. This discrepancy may be attributed to variations in species and treatments. In addition, *MpWOX1a* exhibited a greater increase in expression compared to several other *MpWOX* genes following IAA treatment, likely due to the presence of two auxin responsive binding elements, TGA-element and AuxRR-core, in *MpWOX1a*.There are numerous reports on the response of the ARF gene to auxin. The transcripts *AtARF4*, *AtARF5*, *AtARF16*, *AtARF19*, *OsARF1*, and *OsARF23* exhibited slightly increased expression after auxin treatment (Nagpal et al., 2005; Okushima et al., 2005; Garcia et al., 2006; Wang et al., 2007). The expression of *MpARF* increased after auxin treatment, consistent with previous studies.

## Materials and methods

### Identification of the WOX and ARF genes in *M. pauhoi*

The genome data of *M. pauhoi* used in this study came from local research group of Fujian Normal University. We utilized HMMER 3.3.2, based on the Hidden Markov model files of WOX (PF00046) and ARF (PF06507) from the Pfam (Pfam: Home page (xfam.org), accessed on 15 October 2023), to search for potential WOX and ARF genes in the *M. pauhoi* genome. The potential gene family sequences in *M. pauhoi* were obtained by using BLAST to compare them with the WOX and ARF protein sequences of *A. thaliana* from the PlantTFDB database (PlantTFDB - Plant Transcription Factor Database @ CBI, PKU (gao-lab.org), accessed on 15 October 2023). Finally, the results obtained from HMM search and BLAST(2.5.0) (Altschul et al., 1990) comparison were intersected to obtain the candidate sequences for the final gene family. The candidate proteins were verified to contain the WOX, B3 and Auxin-resp domain using NCBI-CDD (Conserved Domains Database (CDD) and Resources (nih.gov), accessed on 15 October 2023) with the default parameters. Naming of *MpWOX* and *MpARF* genes is based on their homology to *AtWOX* and *AtARF* genes. The WOX and ARF gene families of *Cinnamomum kanehirae* (Chaw et al., 2019), *Phoebe bournei* (Han et al., 2022; Zhang et al., 2023a), *Persea americana* (Nath et al., 2022) and *Cinnamomum camphora* (Shen et al., 2022) were screened and identified using the same method. The physicochemical properties of the gene family were analyzed by ExPASy (http://web.expasy.org/protparam). DeepLoc 2.0 (DeepLoc 2.0 - DTU Health Tech - Bioinformatic Services, accessed on 23 November 2023) was used to predict protein subcellular localization.

### Evolutionary and synteny relationships of the *MpWOX* and *MpARF* genes

The gene sequences of *Oryza sativa* were obtained from the PlantTFDB database. The gene ID and gene name are shown in **Supplementary Table 3**. Phylogenetic trees were constructed using MEGAX (Kumar et al., 2018) (Maximum Likelihood, Bootstrap 1000) and visualized using iTOL (iTOL: Interactive Tree Of Life (embl.de), accessed on 2 November 2023). Gene annotation information was used to map genes on chromosomes. MCScanX (Wang et al., 2012) was used to identify collinearity blocks in *M. pauhoi*. Chromosome localization and collinear results in members of the WOX and ARF gene family are visualized using TBtools.

### The *MpWOX* and *MpARF* gene structures and conserved motifs

The conserved motifs of the gene family proteins were analyzed using MEME (Bailey and Elkan, 1994) (Introduction - MEME Suite (meme-suite.org), accessed on 3 November 2023) (with the following parameter settings: the number of motifs: 10, motif width: 6-100). Phylogenetic relationships, conserved motifs and exon and intron structures of the WOXs and ARFs were visualized using TBtools (Chen et al., 2020). Multiple sequence comparison maps and logo maps of conserved structures of the *M. pauhoi* and *A. thaliana* WOX gene families were drawn using ESPript (https://espript.ibcp.fr/ESPript/cgi-bin/ESPript.cgi) and WebLogo (WebLogo - Create Sequence Logos (berkeley.edu), accessed on 3 November 2023), respectively. SOPMA (https://npsa-prabi.ibcp.fr/cgi-bin/npsa_automat.pl?page=/NPSA/npsa_sopma.html, accessed on 3 November 2023) was used for protein secondary structure prediction. The tertiary structure of protein was predicted using SWISS-MODEL (Waterhouse et al., 2018).

### Cis-acting element analysis of the *MpWOX* and *MpARF* genes

The genome sequence and gene annotation information files were added to the TBtools GFF3 Sequence Extractor submenu. The upstream bases were set to 2000, and the 2000 bp nucleotide sequence upstream of the *MpWOX* and *MpARF* genes were used as the promoter sequence for each *MpWOX* and *MpARF* gene. The promoter cis-regulatory elements of *MpWOX* and *MpARF* genes were predicted using PlantCARE (http://bioinformatics.psb.ugent.be/webtools/plantcare/html/, accessed on 1 November 2023). Excel was used to count cis-elements implicated in significant biological processes such as phytohormone signaling, stress response, site-binding, light responsiveness, and plant growth. The cis-regulatory elements of the *MpWOX* and *MpARF* gene were visualized in Excel.

### The miRNA target prediction and expression pattern analysis of *MpWOXs* and *MpARFs*

The miRNA and target of *MpWOX* and *MpARF* gene were analyzed using psRNATarget (psRNATarget: A Plant Small RNA Target Analysis Server (2017 Update) (zhaolab.org), accessed on 16 November 2023). The transcriptome data used for the study were obtained from our group. The transcriptome data of *M. pauhoi* were obtained from two different lineages, B18 and B28. Among them, B18 lineage plant materials were obtained from Ganzhou, Jiangxi province, and B28 lineage plant materials were provided by Jiande, Zhejiang province. Firstly, all the data were quality controlled and filtered using fastp. The obtained data were aligned to the reference genome using hista2 and the FPKM values of all *MpWOX* and *MpARF* genes were calculated using the FPKM function in the edgeR package. Finally, TBtools was used to visualize the results and generate the expression heatmap of *MpWOX* and *MpARF*. FPKM data Logarithm, set base = 2. Using STRING (https://cn.string-db.org/, accessed on 16 November 2023) protein interaction data, we predicted and constructed protein interaction network.

### Gene expression analysis with RT-qPCR

Plants were grown at Fujian Normal University. We sprayed exogenous hormones to normal growing *M. pauhoi* plants. The hormones consisted of 3-Indoleacetic acid (IAA, 100 μM) and N-1-Naphthylphthalamic acid (NPA, 100 μM). The surface of the plants was sprayed with the atomizer until the leaves are completely wet but without any condensed droplets. Plant leaves were collected in 2.5 mL sterile and enzyme-free cryopreservation tubes before, 6h, 12h and 24h after hormone treatment, respectively, and rapidly frozen in liquid nitrogen. Total RNA was extracted using Plant RNA Kit R6827 and quantified by measuring A260/280 nm and A260/A230 nm values. The obtained RNA was then reverse transcribed into cDNA using Hifair^®^ III 1st Strand cDNA Synthesis SuperMix. Primer sequence information for the *MpWOX* and *MpARF* gene were listed in **Supplementary Table 1**. The RT-qPCR was performed using the *Mpactin* gene as an internal reference gene, and the experiment was set up with three biological replicates. The results were used to calculate the relative gene expression levels using the 2^−ΔΔCt^ method.

## Conclusion

In this study, we identified two gene families related to meristematic tissue growth in five Lauraceae plants, with WOX ranging from 12 to 16 and ARF from 18 to 27. The upstream region of the *MpWOX4* promoter contains two auxin response elements (TGA-elements) and shows a similar expression pattern in the vascular cambium as most *MpARF* genes. *MpWOX4* serve as a conserved gene in the WOX-ARF linkage regulating development in the vascular cambium development.

In addition, *MpWUS*, *MpWOX1a*, and *MpWOX13* was strongly induced by IAA treatment. However, the expression of *MpWOX1a* and *MpWOX13b* was significantly suppressed by NPA treatment. The *MpARF* genes were induced to varying degrees by IAA. Furthermore, there existed a stable linkage between *MpWOX* and *MpARF* genes. Our results will help further investigate the function of *MpWOX* and *MpARF* genes in vascular tissue system, thus providing an important foundation for the cultivation of precious forest resources by genome editing and further synthetic biology.

## Data availability statement

The original M. pauhoi genome and RNA-seq data described in this article have been deposited to NGDC (https://ngdc.cncb.ac.cn/gsub/submit/gsa/list) under the bioproject: PRJCA021595 (CRA013677 and CRA013720). All data generated or analyzed during this study are included in this published article and are also available from the corresponding author on reasonable request.

## Author contributions

MS: Data curation, Investigation, Software, Writing – original draft. KZ: Conceptualization, Funding acquisition, Resources, Supervision, Writing – original draft, Writing – review & editing. XL: Data curation, Formal analysis, Writing – original draft. LG: Resources, Software, Writing – original draft. ZM: Resources, Software, Writing – original draft. LW: Resources, Software, Writing – original draft. SL: Resources, Writing – original draft. YL: Resources, Writing – original draft. HS: Resources, Writing – original draft. SA: Writing – review & editing.
