# Supplementary material for: Genome mining of WOX-ARF gene linkage in Machilus pauhoi underpinned cambial activity associated with IAA induction
Source: Front Plant Sci. 2024 Jul 24;15:1364086. doi: 10.3389/fpls.2024.1364086 (PMC11303294; doi:10.3389/fpls.2024.1364086)
Supplement: Supplementary file 1 [file DataSheet_1.docx]

Figure S1 The distribution of Alpha helix, Beta turn, and Random coil in the secondary structure domains of the MpWOX and MpARF genes proteins


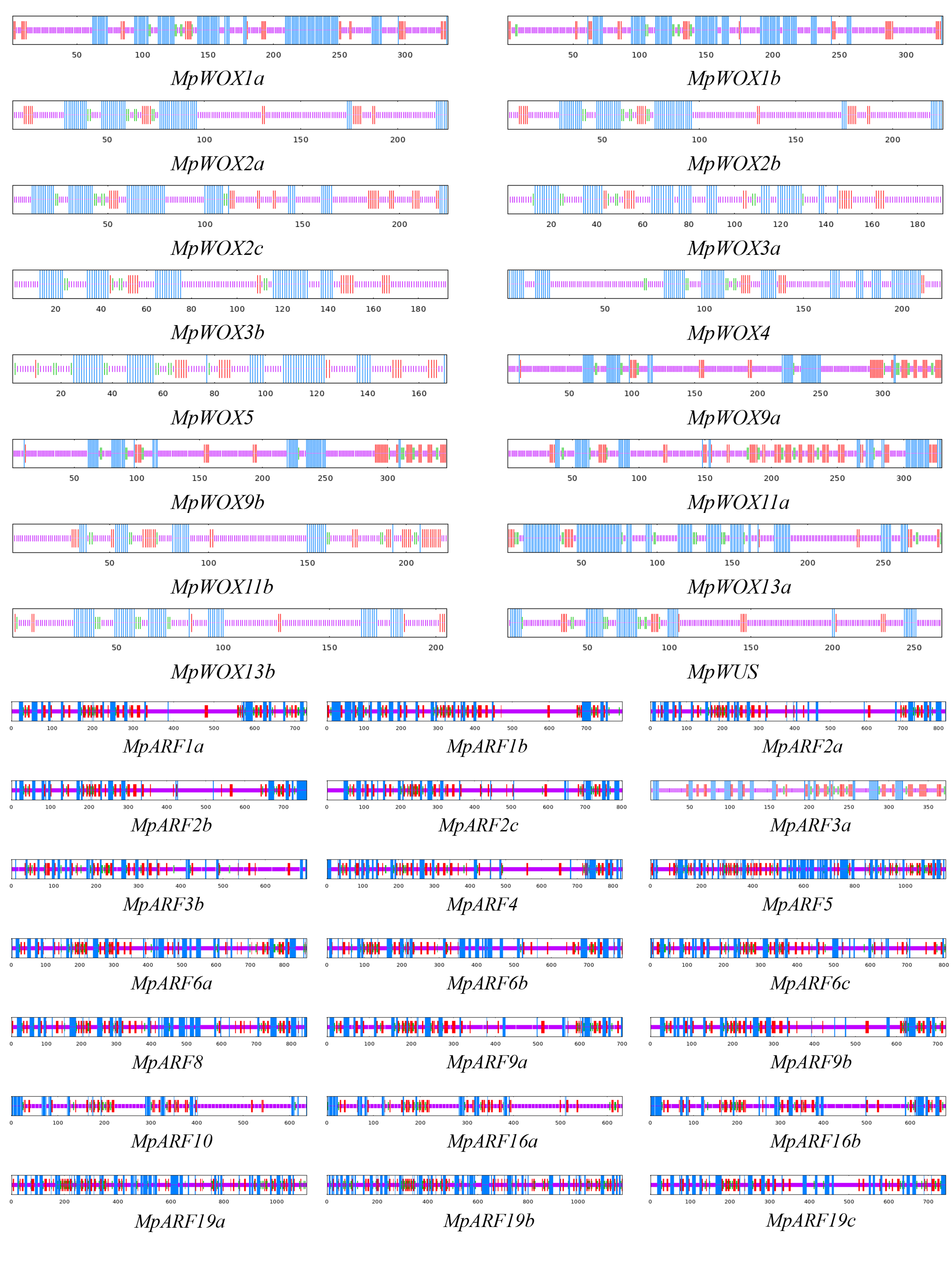


Figure S2 The tertiary structures of the MpWOX and MpARF genes proteins


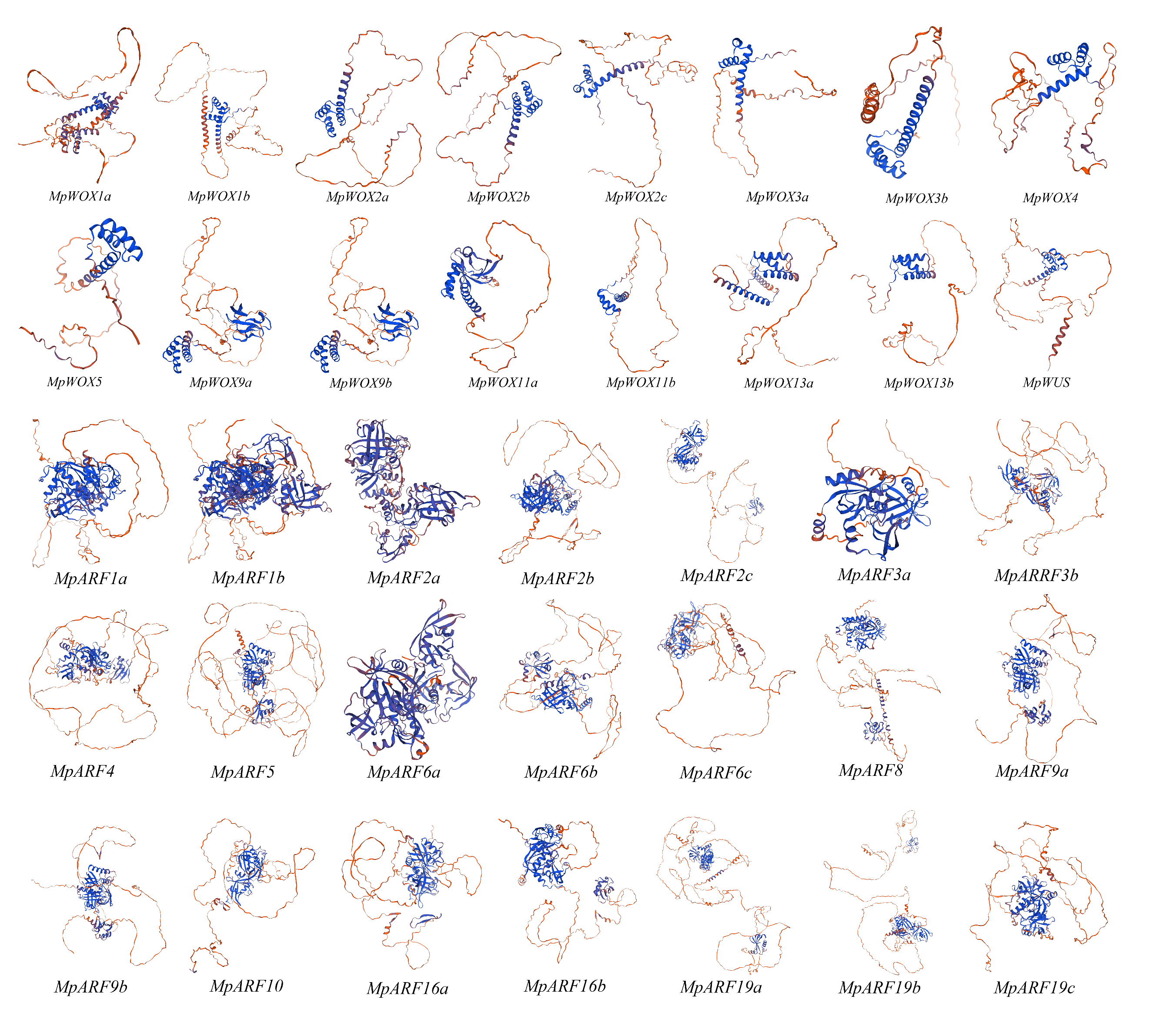


Figure S3 The multiple sequence alignment reveals the presence of the DBD, ARF, and CTD domains in MpARF


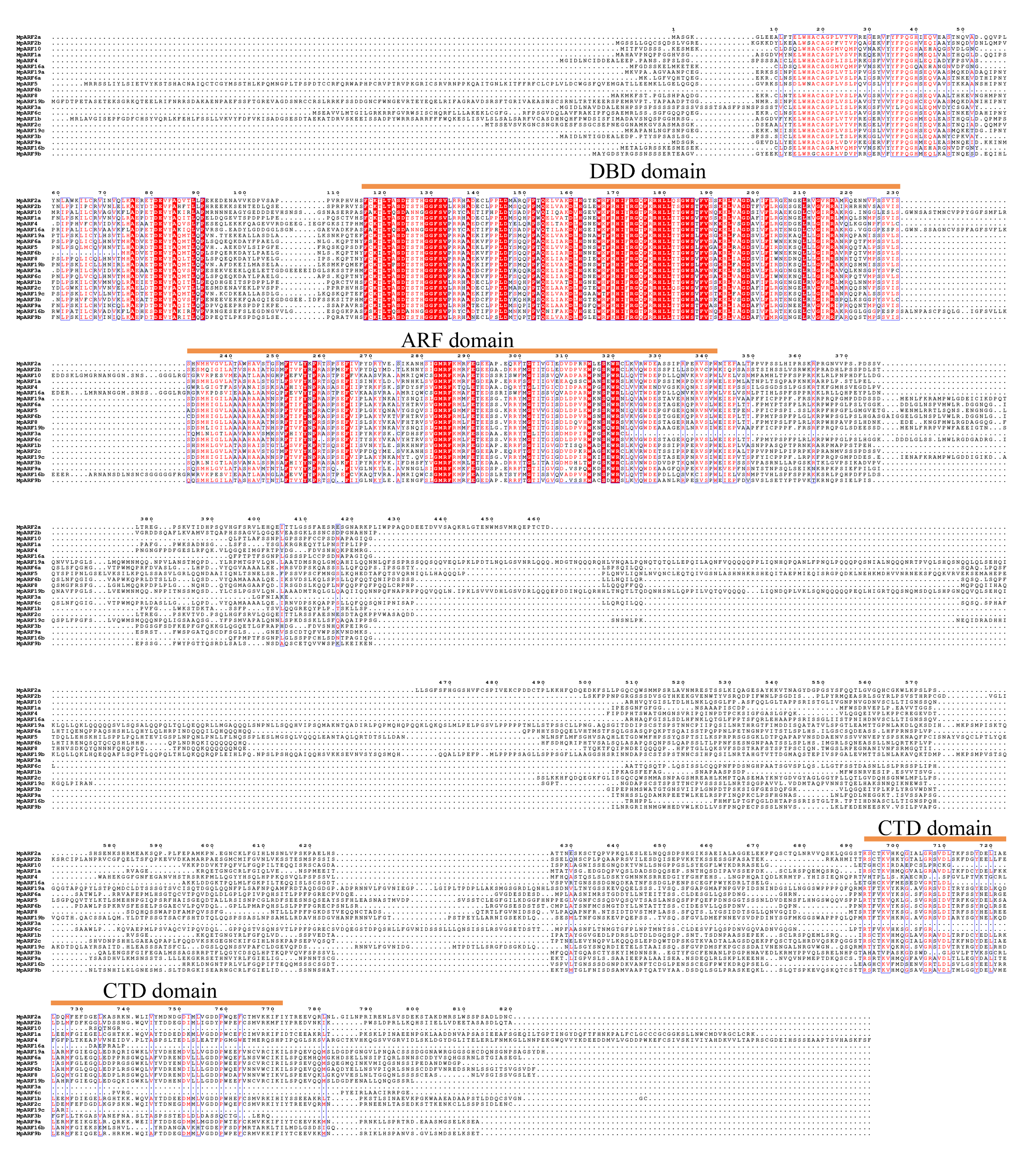


Figure S4 The multiple sequence alignment displays the homeodomain and WUS-box


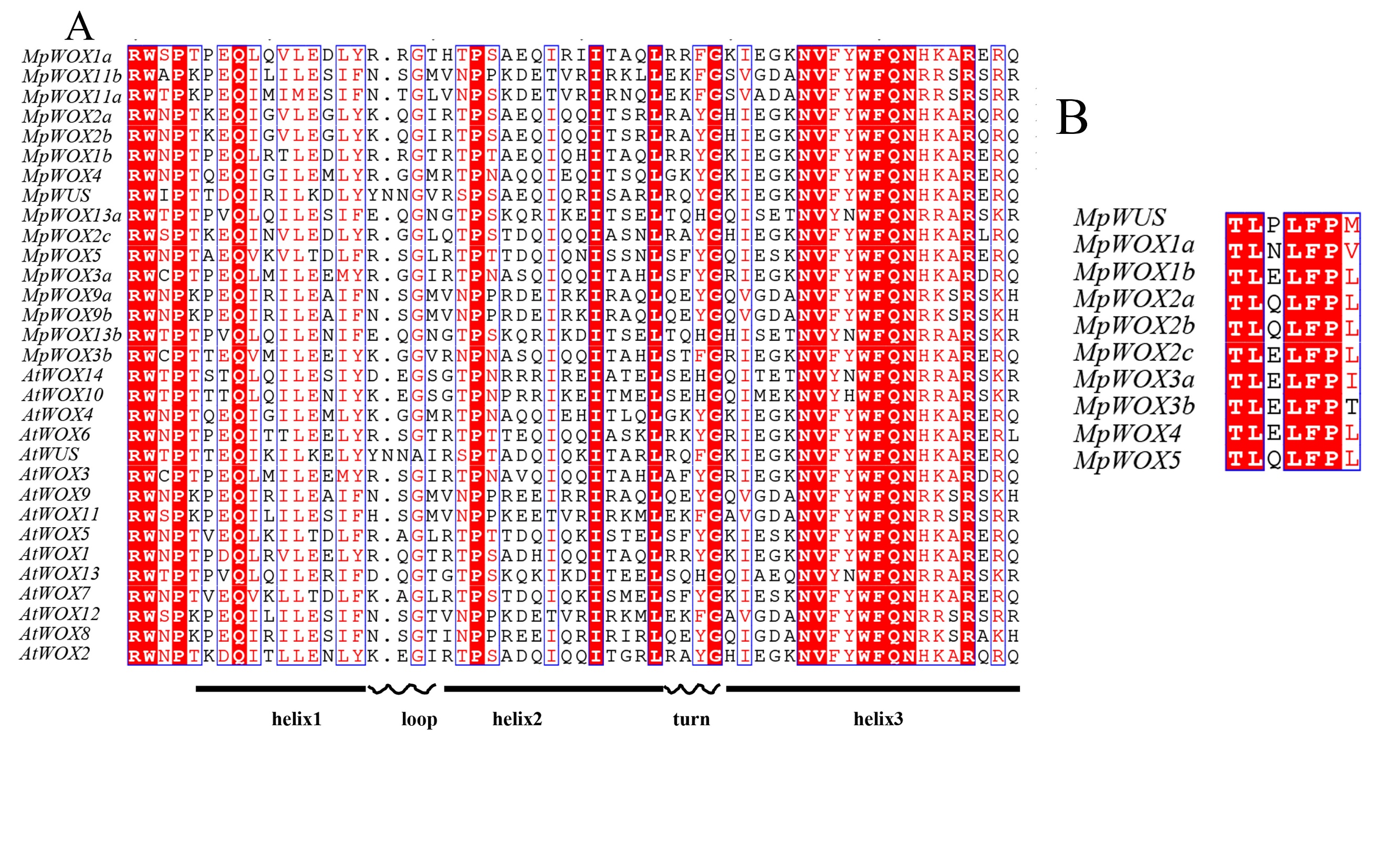


Figure S5 The individual phylogenetic trees of MpWOXs and MpARFs





Table S1 *MpWOX* and *MpARF* primers used for qPCR

| Primer name | Primer sequences |
| --- | --- |
| *Mpactin-F* | AAATAGGACTCCGCTGGCATTGC |
| *Mpactin-R* | GGAAGAGGTTTTGGGGCTCACTG |
| *Mpwox1a-F* | TACTGCTCAACTTCGCCGGTTTG |
| *Mpwox1a-R* | TGCCGCTCCCTCGCCTTG |
| *Mpwox4-F* | ACGTCTCAGCTCGGTAAGTATGGG |
| *Mpwox4-R* | TCCTCTTCTGCCTCTGTCTCTCAC |
| *Mpwus-F* | TCTCCTCACTCAGCTTCCTCTTCC |
| *Mpwus-R* | CTGTTGGTGGTGGTGTTGTTGTTG |
| *Mpwox13a-F* | GTCGTAAGGAGGAGGAGGAGGAAG |
| *Mpwox13a-R* | GCTGCTGCTGATCTCGCTCTTG |
| *Mpwox13b-R* | AGTGGACCTCACCAAAGGGCTAG |
| *Mpwox13b-F* | CCAAACTAGGCTTGCACGAGGAG |
| *MpARF2c-F* | TGTAGGTGAGGTGGGATGAAACTTC |
| *MpARF2c-R* | AGAGGATTAGGCACGGGAGGAG |
| *MpARF1b-F* | CGTTCAGGTTGGTGTGCTTATCTTC |
| *MpARF1b-R* | AAAGGCGTTTGGGCTGCTATTAAC |
| *MpARF6c-F* | ATCTCAATCTCCACACGCCTTCG |
| *MpARF6c-R* | CTGCTGCTGACTGTTGAATGACTG |
| *MpARF4-F* | TCTGTCCAGTTGCCTTCACCATC |
| *MpARF4-R* | ACCGTCCTCTCTTCTGCTCTTATTG |
| *MpARF16a-F* | ACTCTTCTATCGGTGGTCGGAATTG |
| *MpARF16a-R* | TTCTCGCAGTATCTCCAACACAGAC |
| *MpARF2a-F* | GTAAGTGGCGATGATGCGATGAAG |
| *MpARF2a-R* | GAAGACAGAGATTGAACGAGCGAAC |

Table S2 Physicochemical properties and subcellular localization of WOX and ARF protein in *M. pauhoi*

| Gene ID | Gene name | Number of Amino Acid | Molecular Weight | Isoelectric Point | Instability Index | Aliphatic Index | Grand Average of Hydropathicity | Subcellular localization |
| --- | --- | --- | --- | --- | --- | --- | --- | --- |
| BHN000393.1 | *MpWOX1a* | 333 | 37964.66 | 6.61 | 64.15 | 65.89 | -0.669 | nucl |
| BHN000504.1 | *MpWOX11b* | 221 | 23973.88 | 6.73 | 90.56 | 76.74 | -0.337 | nucl |
| BHN007180.1 | *MpWOX11a* | 329 | 36880.7 | 5.76 | 74.51 | 75.23 | -0.46 | nucl |
| BHN009407.1 | *MpWOX2a* | 226 | 25450.57 | 6.18 | 70.3 | 59.96 | -0.721 | nucl |
| BHN009462.1 | *MpWOX2b* | 226 | 25450.57 | 6.18 | 70.3 | 59.96 | -0.721 | nucl |
| BHN010205.1 | *MpWOX1b* | 328 | 26992.41 | 9.13 | 70.59 | 61.95 | -0.722 | nucl |
| BHN010947.1 | *MpWOX4* | 220 | 24813.85 | 8.25 | 56.68 | 62.14 | -0.922 | nucl |
| BHN016561.1 | *MpWUS* | 267 | 30629.91 | 8.86 | 60.61 | 44.23 | -1.038 | nucl |
| BHN017630.1 | *MpWOX13a* | 289 | 32891.85 | 5.97 | 56.04 | 59.34 | -0.975 | nucl |
| BHN022609.1 | *MpWOX2c* | 225 | 25215.48 | 6.83 | 78.25 | 76.71 | -0.418 | nucl |
| BHN022752.1 | *MpWOX5* | 171 | 19864.29 | 7.98 | 60.69 | 72.34 | -0.84 | nucl |
| BHN029814.1 | *MpWOX3a* | 191 | 22181.32 | 9.1 | 57.96 | 59.27 | -0.79 | nucl |
| BHN031174.1 | *MpWOX9a* | 347 | 37754.07 | 8.21 | 55.04 | 57.41 | -0.609 | nucl |
| BHN031225.1 | *MpWOX9b* | 347 | 37754.07 | 8.21 | 55.04 | 57.41 | -0.609 | nucl |
| BHN035396.1 | *MpWOX13b* | 205 | 23092.69 | 6.38 | 62.61 | 53.76 | -0.975 | nucl |
| BHN044469.1 | *MpWOX3b* | 193 | 22757.76 | 9.19 | 59.44 | 52.07 | -0.972 | nucl |
| BHN004173.1 | *MpARF2a* | 820 | 91897.06 | 6.62 | 56.73 | 72.84 | -0.526 | nucl |
| BHN008871.1 | *MpARF2b* | 760 | 84716.11 | 6.74 | 51.91 | 73.47 | -0.419 | nucl |
| BHN012250.1 | *MpARF10* | 637 | 69470.63 | 8.52 | 46.67 | 72.43 | -0.365 | nucl |
| BHN017407.1 | *MpARF1a* | 729 | 80680.09 | 5.5 | 57.07 | 69.95 | -0.438 | nucl |
| BHN017987.1 | *MpARF4* | 827 | 91579.57 | 6.35 | 45.22 | 72.95 | -0.416 | nucl |
| BHN018517.1 | *MpARF16a* | 633 | 68779.68 | 6.48 | 47.5 | 73.35 | -0.349 | nucl |
| BHN020882.1 | *MpARF19a* | 1113 | 123016.72 | 6.07 | 60.22 | 75.55 | -0.52 | nucl |
| BHN022388.1 | *MpARF6a* | 866 | 96522.44 | 6.09 | 64.79 | 75.55 | -0.495 | nucl |
| BHN026802.1 | *MpARF5* | 1158 | 128482.64 | 6.16 | 56.5 | 75.41 | -0.448 | nucl |
| BHN026933.1 | *MpARF6b* | 790 | 87713.03 | 6.45 | 65.98 | 81.33 | -0.387 | nucl |
| BHN029555.1 | *MpARF8* | 845 | 95002.63 | 6.2 | 59.35 | 71.98 | -0.485 | nucl |
| BHN030309.1 | *MpARF19b* | 1177 | 131242.66 | 6.16 | 62.53 | 73.5 | -0.532 | nucl |
| BHN031238.1 | *MpARF3a* | 372 | 41288.75 | 6.64 | 54.08 | 74.97 | -0.343 | nucl |
| BHN032776.1 | *MpARF6c* | 806 | 88423.39 | 8.54 | 70.24 | 78.88 | -0.29 | nucl |
| BHN035769.1 | *MpARF1b* | 798 | 89213.22 | 5.84 | 55.93 | 68.55 | -0.485 | nucl |
| BHN038946.1 | *MpARF2c* | 802 | 88901.88 | 5.85 | 53.94 | 65.75 | -0.588 | nucl |
| BHN044400.1 | *MpARF19c* | 744 | 81580.88 | 6.56 | 56.74 | 73.71 | -0.422 | nucl |
| BHN045480.1 | *MpARF3b* | 698 | 76598.16 | 6.59 | 48.76 | 70.01 | -0.431 | nucl |
| BHN045535.1 | *MpARF9a* | 701 | 78737.33 | 6.17 | 58.67 | 72.74 | -0.515 | nucl |
| BHN045891.1 | *MpARF16b* | 683 | 75145.95 | 6.69 | 51.06 | 70.51 | -0.38 | nucl |
| BHN048040.1 | *MpARF9b* | 720 | 80815.24 | 6.04 | 57.23 | 73.64 | -0.497 | nucl |

Table S3 Sequence information

| gene name | gene ID | gene name | gene ID | gene name | gene ID | gene name | gene ID |
| --- | --- | --- | --- | --- | --- | --- | --- |
| *MpWOX1a* | BHN000393.1 | *AtWOX7* | AT5G05770.1 | *OsARF11* | AL606999 | *CcARF6a* | GWHPBGXC015119 |
| *MpWOX1b* | BHN010205.1 | *AtWOX8* | AT5G45980.1 | *OsARF12* | AL606652 | *CcARF19a* | GWHPBGXC015891 |
| *MpWOX2a* | BHN009407.1 | *AtWOX9* | AT2G33880.1 | *OsARF13* | AL606637 | *CcARF16b* | GWHPBGXC017407 |
| *MpWOX2b* | BHN009462.1 | *AtWUS* | AT2G17950.1 | *OsARF14* | AC132485 | *CcARF5* | GWHPBGXC018048 |
| *MpWOX2c* | BHN022609.1 | *PaWOX1a* | KAJ8637304.1 | *OsARF15* | AC124143 | *CcARF6b* | GWHPBGXC018137 |
| *MpWOX3a* | BHN029814.1 | *PaWOX1b* | KAJ8646691.1 | *OsARF16* | AP003510 | *CcARF19b* | GWHPBGXC020077 |
| *MpWOX3b* | BHN044469.1 | *PaWOX11a* | KAJ8635180.1 | *OsARF17* | AP004989 | *CcARF2b* | GWHPBGXC021716 |
| *MpWOX4* | BHN010947.1 | *PaWOX11b* | KAJ8646592.1 | *OsARF18* | AP005395 | *CcARF6c* | GWHPBGXC023458 |
| *MpWOX5* | BHN022752.1 | *PaWOX13a* | KAJ8625652.1 | *OsARF19* | AP004324 | *CcARF3* | GWHPBGXC024308 |
| *MpWOX9a* | BHN031174.1 | *PaWOX13b* | KAJ8647199.1 | *OsARF20* | AP003861 | *CcARF6d* | GWHPBGXC025838 |
| *MpWOX9b* | BHN031225.1 | *PaWOX2a* | KAJ8636837.1 | *OsARF21* | AP005509 | *CcARF1b* | GWHPBGXC026897 |
| *MpWOX11a* | BHN007180.1 | *PaWOX2b* | KAJ8642340.1 | *OsARF22* | AC024594 | *PaARF1a* | KAJ8625362.1 |
| *MpWOX11b* | BHN000504.1 | *PaWOX3a* | KAJ8620867.1 | *OsARF23* | AC150702 | *PaARF1b* | KAJ8647381.1 |
| *MpWOX13a* | BHN017630.1 | *PaWOX3b* | KAJ8629169.1 | *OsARF24* | AL935066 | *PaARF10* | KAJ8651150.1 |
| *MpWOX13b* | BHN035396.1 | *PaWOX4* | KAJ8637812.1 | *OsARF25* | AL713940 | *PaARF16a* | KAJ8616033.1 |
| *MpWUS* | BHN016561.1 | *PaWOX5* | KAJ8642457.1 | *MpARF2a* | BHN004173.1 | *PaARF16b* | KAJ8639274.1 |
| *CkWOX3a* | CKAN_02486300 | *PaWOX9* | KAJ8626100.1 | *MpARF2b* | BHN008871.1 | *PaARF17* | KAJ8615325.1 |
| *CkWOX9* | CKAN_01869800 | *PaWUS* | KAJ8647965.1 | *MpARF10* | BHN012250.1 | *PaARF19a* | KAJ8628837.1 |
| *CkWOX11a* | CKAN_00420000 | *PbWOX11/12a* | OF22069 | *MpARF1a* | BHN017407.1 | *PaARF19b* | KAJ8641075.1 |
| *CkWOX4* | CKAN_00607200 | *PbWOX11/12b* | OF11766 | *MpARF4* | BHN017987.1 | *PaARF2a* | KAJ8634037.1 |
| *CkWOX2a* | CKAN_00523000 | *PbWOX11/12c* | OF28194 | *MpARF16a* | BHN018517.1 | *PaARF2b* | KAJ8636128.1 |
| *CkWOX1a* | CKAN_00561900 | *PbWOX13a* | OF25757 | *MpARF19a* | BHN020882.1 | *PaARF2c* | KAJ8636490.1 |
| *CkWOX13a* | CKAN_00706200 | *PbWOX13b* | OF14063 | *MpARF6a* | BHN022388.1 | *PaARF2d* | KAJ8644245.1 |
| *CkWUS* | CKAN_00777000 | *PbWOX13c* | OF07768 | *MpARF5* | BHN026802.1 | *PaARF2e* | KAJ8644249.1 |
| *CkWOX3b* | CKAN_01331200 | *PbWOX1a* | OF03970 | *MpARF6b* | BHN026933.1 | *PaARF3a* | KAJ8616405.1 |
| *CkWOX11b* | CKAN_00327500 | *PbWOX1b* | OF11837 | *MpARF8* | BHN029555.1 | *PaARF3b* | KAJ8626112.1 |
| *CkWOX1b* | CKAN_00335600 | *PbWOX2a* | OF19048 | *MpARF19b* | BHN030309.1 | *PaARF3c* | KAJ8633460.1 |
| *CkWOX13b* | CKAN_02217200 | *PbWOX2b* | OF05256 | *MpARF3a* | BHN031238.1 | *PaARF4* | KAJ8638836.1 |
| *CkWOX2b* | CKAN_01032300 | *PbWOX3* | OF16243 | *MpARF6c* | BHN032776.1 | *PaARF5* | KAJ8631531.1 |
| *CkWOX5* | CKAN_01022700 | *PbWOX4* | OF04424 | *MpARF1b* | BHN035769.1 | *PaARF6a* | KAJ8625145.1 |
| *CcWOX1a* | GWHPBGXC000196 | *PbWOX5/7* | OF05362 | *MpARF2c* | BHN038946.1 | *PaARF6b* | KAJ8627250.1 |
| *CcWOX11* | GWHPBGXC000269 | *PbWOX9* | OF24594 | *MpARF19c* | BHN044400.1 | *PaARF6c* | KAJ8631411.1 |
| *CcWUS* | GWHPBGXC010974 | *PbWUS* | OF24054 | *MpARF3b* | BHN045480.1 | *PaARF6d* | KAJ8642156.1 |
| *CcWOX13a* | GWHPBGXC011541 | *AtARF1* | At1g59750 | *MpARF9a* | BHN045535.1 | *PaARF7* | KAJ8621292.1 |
| *CcWOX4* | GWHPBGXC012392 | *AtARF10* | At2g28350 | *MpARF16b* | BHN045891.1 | *PaARF8a* | KAJ8618464.1 |
| *CcWOX1b* | GWHPBGXC012768 | *AtARF11* | At2g46530 | *MpARF9b* | BHN048040.1 | *PaARF8b* | KAJ8629378.1 |
| *CcWOX2a* | GWHPBGXC013079 | *AtARF12* | At1g34310 | *CkARF2b* | CKAN_00502900 | *PaARF9a* | KAJ8616362.1 |
| *CcWOX12* | GWHPBGXC014396 | *AtARF13* | At1g34170 | *CkARF1a* | CKAN_00721000 | *PaARF9b* | KAJ8621816.1 |
| *CcWOX5* | GWHPBGXC014877 | *AtARF14* | At1g35540 | *CkARF6c* | CKAN_01052100 | *PbARF1a* | OF14278 |
| *CcWOX2b* | GWHPBGXC014970 | *AtARF15* | At1g35520 | *CkARF19b* | CKAN_01140300 | *PbARF1b* | OF14279 |
| *CcWOX3* | GWHPBGXC019872 | *AtARF16* | At4g30080 | *CkARF16b* | CKAN_01273700 | *PbARF1c* | OF14282 |
| *CcWOX13b* | GWHPBGXC026663 | *AtARF17* | At1g77850 | *CkARF19a* | CKAN_01302600 | *PbARF10* | OF05709 |
| *OsWUS* | Os04g56780.1 | *AtARF18* | At3g61830 | *CkARF8b* | CKAN_01345200 | *PbARF16a* | OF07141 |
| *OsWOX2* | Os01g62310.1 | *AtARF19* | At1g19220 | *CkARF6b* | CKAN_01471400 | *PbARF16b* | OF14461 |
| *OsWOX3a* | Os11g01130.1 | *AtARF2* | At5g62010 | *CkARF5* | CKAN_01480700 | *PbARF17* | OF13500 |
| *OsWOX3b* | Os05g02730.1 | *AtARF20* | At1g35240 | *CkARF3a* | CKAN_01539700 | *PbARF19a* | OF15166 |
| *OsWOX4* | Os04g55590.1 | *AtARF21* | At1g34410 | *CkARF2a* | CKAN_01585900 | *PbARF19b* | OF23201 |
| *OsWOX5* | Os01g63510.1 | *AtARF22* | At1g34390 | *CkARF6a* | CKAN_01780800 | *PbARF19c* | OF26137 |
| *OsWOX8a* | Os01g47710.1 | *AtARF23* | At1g43950 | *CkARF3b* | CKAN_01868500 | *PbARF2a* | OF02341 |
| *OsWOX8b* | Os07g34880.1 | *AtARF3* | At2g33860 | *CkARF8a* | CKAN_01958500 | *PbARF2b* | OF20735 |
| *OsWOX9* | Os05g48990.1 | *AtARF4* | At5g60450 | *CkARF1b* | CKAN_02193200 | *PbARF2c* | OF21754 |
| *OsWOX11a* | Os07g48560.1 | *AtARF5* | At1g19850 | *CkARF9a* | CKAN_02257900 | *PbARF3a* | OF09049 |
| *OsWOX11b* | Os03g20910.2 | *AtARF6* | At1g30330 | *CkARF7* | CKAN_02521900 | *PbARF3b* | OF12274 |
| *OsWOX12* | Os08g14400.1 | *AtARF7* | At5g20730 | *CkARF3c* | CKAN_02578400 | *PbARF3c* | OF20357 |
| *OsWOX13* | Os01g60270.1 | *AtARF8* | At5g37020 | *CkARF9b* | CKAN_02581900 | *PbARF3d* | OF24604 |
| *AtWOX1* | AT3G18010.1 | *AtARF9* | At4g23980 | *CkARF16a* | CKAN_02605500 | *PbARF4* | OF00399 |
| *AtWOX10* | AT1G20710.1 | *OsARF1* | AP001539 | *CkARF17* | CKAN_02665200 | *PbARF5* | OF01795 |
| *AtWOX11* | AT3G03660.1 | *OsARF2* | AP003706 | *CkARF4* | CKAN_02693300 | *PbARF6a* | OF01686 |
| *AtWOX12* | AT5G17810.1 | *OsARF3* | AP003268 | *CcARF2a* | GWHPBGXC002244 | *PbARF6b* | OF02958 |
| *AtWOX13* | AT4G35550.1 | *OsARF4* | AP004332 | *CcARF11* | GWHPBGXC003874 | *PbARF6c* | OF05094 |
| *AtWOX14* | AT1G20700.1 | *OsARF5* | AP005294 | *CcARF17* | GWHPBGXC006909 | *PbARF6d* | OF15400 |
| *AtWOX2* | AT5G59340.1 | *OsARF6* | AP004863 | *CcARF16a* | GWHPBGXC007644 | *PbARF8a* | OF18244 |
| *AtWOX3* | AT2G28610.1 | *OsARF7* | AP005808 | *CcARF4* | GWHPBGXC007877 | *PbARF8b* | OF22918 |
| *AtWOX4* | AT1G46480.1 | *OsARF8* | AP006168 | *CcARF10* | GWHPBGXC008405 | *PbARF9a* | OF09010 |
| *AtWOX5* | AT3G11260.1 | *OsARF9* | AL662973 | *CcARF1a* | GWHPBGXC011387 | *PbARF9b* | OF14335 |
| *AtWOX6* | AT2G01500.1 | *OsARF10* | AL731629 |  |  |  |  |

Table S4 The protein secondary structure of MpWOX and MpARF protein

| gene name | Alpha helix | Extended strand | Beta turn | Random coil |
| --- | --- | --- | --- | --- |
| *MpWUS* | 17.98% | 6.74% | 4.12% | 71.16% |
| *MpWOX1b* | 28.66% | 9.45% | 3.05% | 58.84% |
| *MpWOX1a* | 33.63% | 9.91% | 2.10% | 54.35% |
| *MpWOX2c* | 31.56% | 12.00% | 3.56% | 52.89% |
| *MpWOX2b* | 24.34% | 8.41% | 3.54% | 63.72% |
| *MpWOX2a* | 24.34% | 8.41% | 3.54% | 63.72% |
| *MpWOX3b* | 28.50% | 9.33% | 3.63% | 58.55% |
| *MpWOX3a* | 31.41% | 9.95% | 4.71% | 53.93% |
| *MpWOX5* | 31.58% | 12.28% | 8.19% | 47.95% |
| *MpWOX4* | 35.00% | 5.00% | 3.64% | 56.36% |
| *MpWOX9b* | 15.56% | 12.39% | 4.32% | 67.72% |
| *MpWOX9a* | 15.56% | 12.39% | 4.32% | 67.72% |
| *MpWOX11b* | 14.48% | 16.74% | 4.98% | 63.80% |
| *MpWOX11a* | 18.24% | 20.36% | 4.56% | 56.84% |
| *MpWOX13b* | 25.37% | 5.85% | 4.39% | 64.39% |
| *MpWOX13a* | 40.48% | 6.92% | 6.23% | 46.37% |
| *MpARF2a* | 19.39% | 14.15% | 3.17% | 63.29% |
| *MpARF2b* | 19.08% | 14.74% | 4.08% | 62.11% |
| *MpARF10* | 15.23% | 12.72% | 3.92% | 68.13% |
| *MpARF1a* | 18.79% | 17.15% | 5.49% | 58.57% |
| *MpARF4* | 19.59% | 14.63% | 4.35% | 61.43% |
| *MpARF16a* | 13.74% | 15.48% | 4.74% | 66.03% |
| *MpARF19a* | 28.03% | 15.72% | 8.72% | 47.53% |
| *MpARF6a* | 26.67% | 14.32% | 4.85% | 54.16% |
| *MpARF5* | 31.87% | 15.63% | 6.39% | 46.11% |
| *MpARF6b* | 25.44% | 13.92% | 3.54% | 57.09% |
| *MpARF8* | 30.06% | 14.56% | 3.55% | 51.83% |
| *MpARF19b* | 29.65% | 15.38% | 8.24% | 46.73% |
| *MpARF3a* | 21.51% | 19.89% | 5.11% | 53.49% |
| *MpARF6c* | 21.09% | 15.76% | 5.09% | 58.06% |
| *MpARF1b* | 24.06% | 16.42% | 4.64% | 54.89% |
| *MpARF2c* | 18.33% | 15.09% | 4.61% | 61.97% |
| *MpARF19c* | 24.33% | 14.78% | 5.78% | 55.11% |
| *MpARF3b* | 16.19% | 13.32% | 5.01% | 65.47% |
| *MpARF9a* | 20.83% | 17.55% | 4.85% | 56.78% |
| *MpARF16b* | 19.77% | 15.37% | 4.54% | 60.32% |
| *MpARF9b* | 20% | 16.67% | 4.17% | 59.17% |

Table S5 Intraspecies and interspecies collinear relationships

|  | Chromosomal localization | gene name | gene ID | Chromosomal localization | gene name | gene ID |
| --- | --- | --- | --- | --- | --- | --- |
| Intraspecies | Chr1 | *MpWOX1a* | BHN000393.1 | Chr2 | *MpWOX1b* | BHN010205.1 |
|  | Chr1 | *MpWOX11b* | BHN000504.1 | Chr2 | *MpWOX11a* | BHN007180.1 |
|  | Chr2 | *MpWOX2b* | BHN009462.1 | Chr4 | *MpWOX2c* | BHN022609.1 |
|  | Chr11 | MpARF16b | BHN045891.1 | Chr3 | *MpARF10* | BHN012250.1 |
|  | Chr11 | *MpARF16b* | BHN045891.1 | Chr4 | *MpARF16a* | BHN018517.1 |
|  | Chr11 | *MpARF3b* | BHN045480.1 | Chr4 | *MpARF4* | BHN017987.1 |
|  | Chr11 | *MpARF3b* | BHN045480.1 | Chr7 | *MpARF3a* | BHN031238.1 |
|  | Chr3 | *MpARF10* | BHN012250.1 | Chr4 | *MpARF16a* | BHN018517.1 |
|  | Chr3 | *MpARF1a* | BHN017407.1 | Chr8 | *MpARF1b* | BHN035769.1 |
|  | Chr4 | *MpARF6a* | BHN022388.1 | Chr6 | *MpARF6b* | BHN026933.1 |
|  | Chr4 | *MpARF19a* | BHN020882.1 | Chr6 | *MpARF19b* | BHN030309.1 |
|  | Chr4 | *MpARF4* | BHN017987.1 | Chr7 | *MpARF3a* | BHN031238.1 |
|  | Chr4 | *MpARF6a* | BHN022388.1 | Chr7 | *MpARF6c* | BHN032776.1 |
| interspecies | Chr3 | *MpWOX13a* | BHN017630.1 | Chr1 | *AtWOX14* | AT1G20700.1 |
|  | Chr3 | *MpWUS* | BHN016561.1 | Chr2 | *AtWUS* | AT2G17950.1 |
|  | Chr3 | *MpWOX13a* | BHN017630.1 | Chr4 | *AtWOX13* | AT4G35550.1 |
|  | Chr7 | *MpWOX9b* | BHN031225.1 | Chr5 | *AtWOX8* | AT5G45980.1 |
|  | Chr8 | *MpWOX13b* | BHN035395.1 | Chr1 | *AtWOX14* | AT1G20700.1 |
|  | Chr8 | *MpWOX13b* | BHN035395.1 | Chr4 | *AtWOX13* | AT4G35550.1 |
|  | Chr3 | *MpARF10* | BHN012250.1 | Chr2 | *AtARF10* | AT2G28350.1 |
|  | Chr4 | *MpARF6a* | BHN022388.1 | Chr1 | *AtARF5* | AT1G19850.1 |
|  | Chr6 | *MpARF5* | BHN026802.1 | Chr1 | *AtARF5* | AT1G19850.1 |
|  | Chr10 | *MpARF19c* | BHN044400.1 | Chr1 | *AtARF19* | AT1G19220.1 |
|  | Chr10 | *MpARF19c* | BHN044400.1 | Chr5 | *AtARF7* | AT5G20730.1 |
|  | Chr11 | *MpARF16b* | BHN045891.1 | Chr2 | *AtARF10* | AT2G28350.1 |
|  | Chr11 | *MpARF3b* | BHN045480.1 | Chr2 | *AtARF3* | AT2G33860.1 |
|  | Chr11 | *MpARF9a* | BHN045535.1 | Chr3 | *AtARF18* | AT3G61830.1 |
|  | Chr11 | *MpARF9a* | BHN045535.1 | Chr4 | *AtARF9* | AT4G23980.1 |

Table S6 The predicted target sites of miRNA of *MpWOX* and *MpARF* by psRNATarget

| miRNA_Acc. | Gene name | miRNA_start | miRNA_end | Target_start | Target_end | miRNA_aligned_fragment | Target_aligned_fragment |
| --- | --- | --- | --- | --- | --- | --- | --- |
| ath-miR1888a | *MpWOX11a* | 1 | 21 | 536 | 556 | UAAGUUAAGAUUUGUGAAGAA | UACAUUACCAAUCUGGACUUA |
| ath-miR4239 | *MpWOX11a* | 1 | 21 | 153 | 173 | UUUGUUAUUUUCGCAUGCUCC | UGAGACUGUGAGGAUAAGAAA |
| ath-miR5016 | *MpWOX11a* | 1 | 21 | 460 | 480 | UUCUUGUGGAUUCCUUGGAAA | CAGCAAAUGGGUUCGCAAGAA |
| ath-miR781a | *MpWOX11a* | 1 | 21 | 746 | 766 | UUAGAGUUUUCUGGAUACUUA | CUCGAAUGCAGAAAAUUCCAA |
| ath-miR781b | *MpWOX11a* | 1 | 21 | 746 | 766 | UUAGAGUUUUCUGGAUACUUA | CUCGAAUGCAGAAAAUUCCAA |
| ath-miR5021 | *MpWOX11b* | 1 | 20 | 352 | 371 | UGAGAAGAAGAAGAAGAAAA | UUUGCUUCUCCUUCUUCUUC |
| ath-miR825 | *MpWOX11b* | 1 | 21 | 159 | 179 | UUCUCAAGAAGGUGCAUGAAC | GAUAAGGAAGCUUCUUGAGAA |
| ath-miR426 | *MpWOX13a* | 1 | 21 | 431 | 451 | UUUUGGAAAUUUGUCCUUACG | AACAUGGCCAAAUUUCUGAAA |
| ath-miR5651 | *MpWOX13a* | 1 | 21 | 198 | 218 | UUGUGCGGUUCAAAUAGUAAC | GCAGCUAGUUGAGUUGCACAA |
| ath-miR8170-3p | *MpWOX13a* | 1 | 21 | 355 | 375 | UUGCUUAAAGAUUUUCUAUGU | CUUGAGAGUAUCUUUGAGCAA |
| ath-miR838 | *MpWOX13a* | 1 | 21 | 36 | 56 | UUUUCUUCUACUUCUUGCACA | GGAGGAGGAGGAAGAAGAAAA |
| ath-miR158a-5p | *MpWOX13b* | 1 | 21 | 278 | 297 | CUUUGUCUACAAUUUUGGAAA | AAUCUGAAGUUG-AGACAGAG |
| ath-miR5629 | *MpWOX1a* | 1 | 22 | 532 | 553 | UUAGGGUAGUUAACGGAAGUUA | AAAUUUUUGUUAACUCCCCAAG |
| ath-miR865-3p | *MpWOX1a* | 1 | 21 | 376 | 396 | UUUUUCCUCAAAUUUAUCCAA | UUUGGUAAGAUUGAGGGGAAG |
| ath-miR5658 | *MpWOX1b* | 1 | 21 | 125 | 145 | AUGAUGAUGAUGAUGAUGAAA | GAUCAUCUUCGUCAUCCUCUU |
| ath-miR776 | *MpWOX1b* | 1 | 21 | 808 | 827 | UCUAAGUCUUCUAUUGAUGUU | CCAAUCAAUGGAAG-CUUGGA |
| ath-miR860 | *MpWOX1b* | 1 | 21 | 799 | 819 | UCAAUAGAUUGGACUAUGUAU | UCAAAUAUUCCAAUCAAUGGA |
| ath-miR862-3p | *MpWOX1b* | 1 | 21 | 336 | 356 | AUAUGCUGGAUCUACUUGAAG | UGCCGAACAGAUCCAGCACAU |
| ath-miR5642a | *MpWOX2a* | 1 | 20 | 108 | 127 | UCUCGCGCUUGUACGGCUUU | GGGGCUGUACAAGCAAGGGA |
| ath-miR5642b | *MpWOX2a* | 1 | 20 | 108 | 127 | UCUCGCGCUUGUACGGCUUU | GGGGCUGUACAAGCAAGGGA |
| ath-miR5642a | *MpWOX2b* | 1 | 20 | 108 | 127 | UCUCGCGCUUGUACGGCUUU | GGGGCUGUACAAGCAAGGGA |
| ath-miR5642b | *MpWOX2b* | 1 | 20 | 108 | 127 | UCUCGCGCUUGUACGGCUUU | GGGGCUGUACAAGCAAGGGA |
| ath-miR833a-5p | *MpWOX2c* | 1 | 22 | 90 | 111 | UGUUUGUUGUACUCGGUCUAGU | CACAGAUCAGAUACAGCAGAUA |
| ath-miR865-5p | *MpWOX2c* | 1 | 21 | 309 | 329 | AUGAAUUUGGAUCUAAUUGAG | UGCAAAAAGGUCUGAGUUUGU |
| ath-miR164b-3p | *MpWOX3a* | 1 | 21 | 438 | 458 | CAUGUGCCCAUCUUCACCAUC | GGUGGUGAUGAUGGAUGUGUG |
| ath-miR414 | *MpWOX3a* | 1 | 21 | 437 | 457 | UCAUCUUCAUCAUCAUCGUCA | GGGUGGUGAUGAUGGAUGUGU |
| ath-miR838 | *MpWOX4* | 1 | 21 | 595 | 614 | UUUUCUUCUACUUCUUGCACA | GGUG-AAGAAGAAGAGGAGGA |
| ath-miR838 | *MpWOX4* | 1 | 21 | 540 | 560 | UUUUCUUCUACUUCUUGCACA | AAUUCAGAGGGUGGAAGAGGA |
| ath-miR847 | *MpWOX4* | 1 | 21 | 595 | 615 | UCACUCCUCUUCUUCUUGAUG | GGUGAAGAAGAAGAGGAGGAG |
| ath-miR773a | *MpWOX5* | 1 | 21 | 28 | 48 | UUUGCUUCCAGCUUUUGUCUC | GUGAAAGGAGUUGGAGGGAAA |
| ath-miR838 | *MpWOX5* | 1 | 21 | 27 | 47 | UUUUCUUCUACUUCUUGCACA | UGUGAAAGGAGUUGGAGGGAA |
| ath-miR865-3p | *MpWOX5* | 1 | 21 | 385 | 405 | UUUUUCCUCAAAUUUAUCCAA | CUGAAUACGUAUGAGGAGGAA |
| ath-miR2934-5p | *MpWOX9a* | 1 | 21 | 925 | 945 | UCUUUCUGCAAACGCCUUGGA | CGCGAGGCGUUUGGAGAAGAG |
| ath-miR5641 | *MpWOX9a* | 1 | 21 | 380 | 400 | UGGAAGAAGAUGAUAGAAUUA | CAGUCUCUUCAUCUUCUUCCU |
| ath-miR8181 | *MpWOX9a* | 1 | 20 | 947 | 965 | UGGGGGUGGGGGGGUGACAG | CUGUC-CUCCUCCACUCCUC |
| ath-miR8181 | *MpWOX9a* | 1 | 20 | 575 | 594 | UGGGGGUGGGGGGGUGACAG | GUGGAACCCUCUCAUCCCAA |
| ath-miR870-3p | *MpWOX9a* | 1 | 21 | 465 | 485 | UAAUUUGGUGUUUCUUCGAUC | GAUCGAAGUAGCAUCCAAUUC |
| ath-miR2934-5p | *MpWOX9b* | 1 | 21 | 925 | 945 | UCUUUCUGCAAACGCCUUGGA | CGCGAGGCGUUUGGAGAAGAG |
| ath-miR5641 | *MpWOX9b* | 1 | 21 | 380 | 400 | UGGAAGAAGAUGAUAGAAUUA | CAGUCUCUUCAUCUUCUUCCU |
| ath-miR8181 | *MpWOX9b* | 1 | 20 | 947 | 965 | UGGGGGUGGGGGGGUGACAG | CUGUC-CUCCUCCACUCCUC |
| ath-miR8181 | *MpWOX9b* | 1 | 20 | 575 | 594 | UGGGGGUGGGGGGGUGACAG | GUGGAACCCUCUCAUCCCAA |
| ath-miR870-3p | *MpWOX9b* | 1 | 21 | 465 | 485 | UAAUUUGGUGUUUCUUCGAUC | GAUCGAAGUAGCAUCCAAUUC |
| ath-miR5658 | *MpWUS* | 1 | 21 | 43 | 63 | AUGAUGAUGAUGAUGAUGAAA | CAGCAGCAGCAUCAUCAACAU |
| ath-miR838 | *MpWUS* | 1 | 21 | 296 | 316 | UUUUCUUCUACUUCUUGCACA | CAAGGGAGAGGCAGAAGAAGA |
| ath-miR863-5p | *MpWUS* | 1 | 21 | 490 | 510 | UUAUGUCUUGUUGAUCUCAAU | GGUGGGAACAACGUGGCAUGG |
| ath-miR160a-5p | *MpARF2a* | 1 | 21 | 1238 | 1258 | UGCCUGGCUCCCUGUAUGCCA | CUGAAAGUAGAGAGUCAGGCA |
| ath-miR160b | *MpARF2a* | 1 | 21 | 1238 | 1258 | UGCCUGGCUCCCUGUAUGCCA | CUGAAAGUAGAGAGUCAGGCA |
| ath-miR160c-5p | *MpARF2a* | 1 | 21 | 1238 | 1258 | UGCCUGGCUCCCUGUAUGCCA | CUGAAAGUAGAGAGUCAGGCA |
| ath-miR164a | *MpARF2a* | 1 | 21 | 1418 | 1437 | UGGAGAAGCAGGGCACGUGCA | CACAUGUGUUUUG-UUCUCCA |
| ath-miR164b-5p | *MpARF2a* | 1 | 21 | 1418 | 1437 | UGGAGAAGCAGGGCACGUGCA | CACAUGUGUUUUG-UUCUCCA |
| ath-miR164c-5p | *MpARF2a* | 1 | 21 | 1418 | 1437 | UGGAGAAGCAGGGCACGUGCG | CACAUGUGUUUUG-UUCUCCA |
| ath-miR3932a | *MpARF2a* | 1 | 21 | 2121 | 2141 | AACUUUGUGAUGACAACGAAG | GUCUGUUGACCUUACAAAGUU |
| ath-miR3932b-3p | *MpARF2a* | 1 | 21 | 2121 | 2141 | AACUUUGUGAUGACAACGAAG | GUCUGUUGACCUUACAAAGUU |
| ath-miR395a | *MpARF2a* | 1 | 21 | 499 | 519 | CUGAAGUGUUUGGGGGAACUC | CGAUUCCGCCAUAUAUUUCGG |
| ath-miR395d | *MpARF2a* | 1 | 21 | 499 | 519 | CUGAAGUGUUUGGGGGAACUC | CGAUUCCGCCAUAUAUUUCGG |
| ath-miR395e | *MpARF2a* | 1 | 21 | 499 | 519 | CUGAAGUGUUUGGGGGAACUC | CGAUUCCGCCAUAUAUUUCGG |
| ath-miR5649a | *MpARF2a* | 1 | 21 | 1365 | 1385 | AUUGAAUAUGUUGGUUACUAU | GCAGGAGCCAACAUGUACAGA |
| ath-miR5649b | *MpARF2a* | 1 | 21 | 1365 | 1385 | AUUGAAUAUGUUGGUUACUAU | GCAGGAGCCAACAUGUACAGA |
| ath-miR773b-3p | *MpARF2a* | 1 | 21 | 936 | 956 | UUUGAUUCCAGCUUUUGUCUC | CAGGAGAUGGCUUGAAUCAAA |
| ath-miR858b | *MpARF2a* | 1 | 21 | 222 | 242 | UUCGUUGUCUGUUCGACCUUG | GAAGGCGGAACGGGAAACGGA |
| ath-miR159a | *MpARF2b* | 1 | 21 | 1621 | 1641 | UUUGGAUUGAAGGGAGCUCUA | UUGACAUCUUUUCAACCCAAG |
| ath-miR159b-3p | *MpARF2b* | 1 | 21 | 1621 | 1641 | UUUGGAUUGAAGGGAGCUCUU | UUGACAUCUUUUCAACCCAAG |
| ath-miR159c | *MpARF2b* | 1 | 21 | 1621 | 1641 | UUUGGAUUGAAGGGAGCUCCU | UUGACAUCUUUUCAACCCAAG |
| ath-miR2112-3p | *MpARF2b* | 1 | 21 | 922 | 942 | CUUUAUAUCCGCAUUUGCGCA | GAAGAAGGUGCGGAUAAAAGG |
| ath-miR414 | *MpARF2b* | 1 | 21 | 2129 | 2149 | UCAUCUUCAUCAUCAUCGUCA | GCUCAAUGGUGAGGAAGAUGU |
| ath-miR865-5p | *MpARF2b* | 1 | 21 | 783 | 803 | AUGAAUUUGGAUCUAAUUGAG | UGCAACUGGGUCCAUGUUCAC |
| ath-miR5018 | *MpARF10* | 1 | 24 | 824 | 847 | UUAAAGCUCCACCAUGAGUCCAAU | AUUCAAAUUCUGGUGGGGGUUUGA |
| ath-miR5641 | *MpARF10* | 1 | 21 | 615 | 635 | UGGAAGAAGAUGAUAGAAUUA | GGACUCAAUUGUCUUCUUGCG |
| ath-miR8181 | *MpARF10* | 1 | 20 | 1481 | 1500 | UGGGGGUGGGGGGGUGACAG | GUCUCACUGCUCCACCCUCC |
| ath-miR862-3p | *MpARF10* | 1 | 21 | 516 | 536 | AUAUGCUGGAUCUACUUGAAG | GGUUUGGAAGUUCCGGCAUAU |
| ath-miR172d-5p | *MpARF1a* | 1 | 21 | 894 | 914 | GCAACAUCUUCAAGAUUCAGA | GAUGAGAUUUGAAGGUGAUGA |
| ath-miR2111a-5p | *MpARF1a* | 1 | 21 | 1530 | 1550 | UAAUCUGCAUCCUGAGGUUUA | UCAAUCUUUGGAUGCAGACUC |
| ath-miR2111b-5p | *MpARF1a* | 1 | 21 | 1530 | 1550 | UAAUCUGCAUCCUGAGGUUUA | UCAAUCUUUGGAUGCAGACUC |
| ath-miR414 | *MpARF1a* | 1 | 21 | 1818 | 1838 | UCAUCUUCAUCAUCAUCGUCA | CUACACAGAUGAUGAAGAUGA |
| ath-miR825 | *MpARF1a* | 1 | 21 | 626 | 646 | UUCUCAAGAAGGUGCAUGAAC | AUGCAUUUAUCUUCUUAAGAG |
| ath-miR835-5p | *MpARF1a* | 1 | 21 | 1672 | 1692 | UUCUUGCAUAUGUUCUUUAUC | ACCAAGGUACACAUGCAAGGG |
| ath-miR853 | *MpARF1a* | 1 | 22 | 1206 | 1227 | UCCCCUCUUUAGCUUGGAGAAG | AUAUUCUGGGCUUAAGAGAGGA |
| ath-miR172e-3p | *MpARF4* | 1 | 21 | 1232 | 1252 | GGAAUCUUGAUGAUGCUGCAU | UUGGGGAAUCUUUAAGGUUCC |
| ath-miR1886.2 | *MpARF4* | 1 | 21 | 2463 | 2483 | UGAGAUGAAAUCUUUGAUUGG | UGCAUCCAAGUUUUCAUUUUG |
| ath-miR5020b | *MpARF4* | 1 | 21 | 1664 | 1684 | AUGGCAUGAAAGAAGGUGAGA | CUUCACCAUCUUCUGUGCUAA |
| ath-miR832-5p | *MpARF4* | 1 | 21 | 1343 | 1363 | UGCUGGGAUCGGGAAUCGAAA | GAUUCAUUCCUGAUCCCCACA |
| ath-miR396b-3p | *MpARF16a* | 1 | 21 | 607 | 627 | GCUCAAGAAAGCUGUGGGAAA | GAUUCAAUAGUGUUCUUGAGA |
| ath-miR406 | *MpARF16a* | 1 | 21 | 604 | 623 | UAGAAUGCUAUUGUAAUCCAG | GGUGAUU-CAAUAGUGUUCUU |
| ath-miR839-5p | *MpARF16a* | 1 | 21 | 763 | 783 | UACCAACCUUUCAUCGUUCCC | AAGGAAGAUGAAAGGCUGAUG |
| ath-miR3932a | *MpARF19a* | 1 | 21 | 229 | 249 | AACUUUGUGAUGACAACGAAG | AUUUGUUAUCUUCACAGUGUU |
| ath-miR3932b-3p | *MpARF19a* | 1 | 21 | 229 | 249 | AACUUUGUGAUGACAACGAAG | AUUUGUUAUCUUCACAGUGUU |
| ath-miR399c-5p | *MpARF19a* | 1 | 21 | 598 | 618 | GGGCAUCUUUCUAUUGGCAGG | UUUGUCAGUGGAAAGAGACUC |
| ath-miR417 | *MpARF19a* | 1 | 21 | 221 | 241 | GAAGGUAGUGAAUUUGUUCGA | CAAAAUUGAUUUGUUAUCUUC |
| ath-miR5023 | *MpARF19a* | 1 | 21 | 2548 | 2568 | AUUGGUAGUGGAUAAGGGGGC | UUCCCAUUAUCAGCUUUCAAU |
| ath-miR8181 | *MpARF19a* | 1 | 20 | 2108 | 2127 | UGGGGGUGGGGGGGUGACAG | CAGUGCUUCCUCCACCUCCA |
| ath-miR841a-3p | *MpARF19a* | 1 | 21 | 2261 | 2281 | AUUUCUAGUGGGUCGUAUUCA | UAAAUAGGACCCACAGGGGAA |
| ath-miR824-5p | *MpARF6a* | 1 | 21 | 559 | 579 | UAGACCAUUUGUGAGAAGGGA | CAUCUUCUCACAACUGGUUGG |
| ath-miR159a | *MpARF5* | 1 | 21 | 319 | 339 | UUUGGAUUGAAGGGAGCUCUA | UGGGGUUCUUUUCAAGUUGAA |
| ath-miR159a | *MpARF5* | 1 | 21 | 1140 | 1160 | UUUGGAUUGAAGGGAGCUCUA | UACAGUUUUCUAUAAUCCAAG |
| ath-miR159b-3p | *MpARF5* | 1 | 21 | 319 | 339 | UUUGGAUUGAAGGGAGCUCUU | UGGGGUUCUUUUCAAGUUGAA |
| ath-miR159b-3p | *MpARF5* | 1 | 21 | 1140 | 1160 | UUUGGAUUGAAGGGAGCUCUU | UACAGUUUUCUAUAAUCCAAG |
| ath-miR159c | *MpARF5* | 1 | 21 | 319 | 339 | UUUGGAUUGAAGGGAGCUCCU | UGGGGUUCUUUUCAAGUUGAA |
| ath-miR159c | *MpARF5* | 1 | 21 | 1140 | 1160 | UUUGGAUUGAAGGGAGCUCCU | UACAGUUUUCUAUAAUCCAAG |
| ath-miR169b-3p | *MpARF5* | 1 | 22 | 3225 | 3246 | GGCAAGUUGUCCUUCGGCUACA | UGGACUUGAAGGGCAACUUGAU |
| ath-miR3932a | *MpARF5* | 1 | 21 | 3162 | 3182 | AACUUUGUGAUGACAACGAAG | GUCCAUUGAUGUCACAAGGUU |
| ath-miR3932b-3p | *MpARF5* | 1 | 21 | 3162 | 3182 | AACUUUGUGAUGACAACGAAG | GUCCAUUGAUGUCACAAGGUU |
| ath-miR5013 | *MpARF5* | 1 | 21 | 3159 | 3179 | UUUGUGACAUCUAGGUGCUUU | GAGGUCCAUUGAUGUCACAAG |
| ath-miR5631 | *MpARF5* | 1 | 21 | 3290 | 3310 | UGGCAGGAAAGACAUAAUUUU | AAAAUGAUGUUCUUCUUGUUG |
| ath-miR8168 | *MpARF5* | 1 | 21 | 755 | 775 | AGGUGCUGAGUGUGCUAGUGC | AUACCAGCACACACGGCGGCU |
| ath-miR8181 | *MpARF5* | 1 | 20 | 1443 | 1462 | UGGGGGUGGGGGGGUGACAG | UUGUCCCUCUCCCAUUUCAA |
| ath-miR837-5p | *MpARF5* | 1 | 21 | 1402 | 1422 | AUCAGUUUCUUGUUCGUUUCA | GUUAACGUAUGGGAAAUUGAG |
| ath-miR846-3p | *MpARF5* | 1 | 21 | 58 | 78 | UUGAAUUGAAGUGCUUGAAUU | CAUGCAUGCAAUGCAAUUCAA |
| ath-miR863-5p | *MpARF5* | 1 | 21 | 2714 | 2734 | UUAUGUCUUGUUGAUCUCAAU | UAUCAGGUGAACAAGACACAG |
| ath-miR870-3p | *MpARF5* | 1 | 21 | 1187 | 1207 | UAAUUUGGUGUUUCUUCGAUC | CUUUGGGCAAAUACCAAAAUG |
| ath-miR173-3p | *MpARF6b* | 1 | 21 | 478 | 498 | UGAUUCUCUGUGUAAGCGAAA | GAUAGCAUGCACAUAGGAUUA |
| ath-miR396b-3p | *MpARF6b* | 1 | 21 | 1401 | 1421 | GCUCAAGAAAGCUGUGGGAAA | UCUUUCUCAGCUUGCUUCAGC |
| ath-miR5629 | *MpARF6b* | 1 | 22 | 1825 | 1846 | UUAGGGUAGUUAACGGAAGUUA | UGCAUGCCGUUAGCUACUUCAA |
| ath-miR833b | *MpARF6b* | 1 | 21 | 1803 | 1822 | UGUUUGUUGACAUCGGUCUAG | UGAGUCUGAU-UCAACAAGCA |
| ath-miR5014a-5p | *MpARF8* | 1 | 21 | 2251 | 2271 | ACACUUAGUUUUGUACAACAU | CAGAUGUAUGGAAUUGAGGGU |
| ath-miR5649a | *MpARF8* | 1 | 21 | 1298 | 1318 | AUUGAAUAUGUUGGUUACUAU | UAGGAAAUCAGCAUGAUCAGU |
| ath-miR5649b | *MpARF8* | 1 | 21 | 1298 | 1318 | AUUGAAUAUGUUGGUUACUAU | UAGGAAAUCAGCAUGAUCAGU |
| ath-miR5653 | *MpARF8* | 1 | 24 | 283 | 306 | UGGGUUGAGUUGAGUUGAGUUGGC | GAAGUCUAUGCUCAACUCACCCUG |
| ath-miR840-5p | *MpARF8* | 1 | 22 | 1238 | 1259 | ACACUGAAGGACCUAAACUAAC | UGGGUUUUAGGUCUUUUGGUUU |
| ath-miR2938 | *MpARF19b* | 1 | 21 | 1536 | 1556 | GAUCUUUUGAGAGGGUUCCAG | GGAGAACCUUUUCAGGAGGCC |
| ath-miR3440b-3p | *MpARF19b* | 1 | 21 | 1918 | 1938 | UGGAUUGGUCAAGGGAAGCGU | AGGCAUCAUUUGACCAAUCAG |
| ath-miR395b | *MpARF19b* | 1 | 21 | 3448 | 3468 | CUGAAGUGUUUGGGGGGACUC | UUGUCCCCUCAAGAAGUUCAA |
| ath-miR395c | *MpARF19b* | 1 | 21 | 3448 | 3468 | CUGAAGUGUUUGGGGGGACUC | UUGUCCCCUCAAGAAGUUCAA |
| ath-miR395f | *MpARF19b* | 1 | 21 | 3448 | 3468 | CUGAAGUGUUUGGGGGGACUC | UUGUCCCCUCAAGAAGUUCAA |
| ath-miR406 | *MpARF19b* | 1 | 21 | 2330 | 2350 | UAGAAUGCUAUUGUAAUCCAG | AAGCAAUCCAACAGCAUUCUG |
| ath-miR426 | *MpARF19b* | 1 | 21 | 1578 | 1598 | UUUUGGAAAUUUGUCCUUACG | AAUUGGCACAAAUUCUCAGAA |
| ath-miR853 | *MpARF3a* | 1 | 22 | 310 | 331 | UCCCCUCUUUAGCUUGGAGAAG | AAGCUCCAUGCUGAGGCGGCGA |
| ath-miR156i | *MpARF6c* | 1 | 20 | 275 | 294 | UGACAGAAGAGAGAGAGCAG | GUGCUGGCCCUCUUGUGUCA |
| ath-miR156j | *MpARF6c* | 1 | 20 | 275 | 294 | UGACAGAAGAGAGAGAGCAC | GUGCUGGCCCUCUUGUGUCA |
| ath-miR418 | *MpARF6c* | 1 | 21 | 1006 | 1026 | UAAUGUGAUGAUGAACUGACC | UCUGAGUUUGUCAUUACAUAU |
| ath-miR780.1 | *MpARF6c* | 1 | 21 | 213 | 233 | UCUAGCAGCUGUUGAGCAGGU | CUUUGGUCAACAGCCGCAGGA |
| ath-miR833b | *MpARF6c* | 1 | 21 | 2139 | 2158 | UGUUUGUUGACAUCGGUCUAG | UGAGACUGAU-UCAACAACCA |
| ath-miR835-3p | *MpARF6c* | 1 | 21 | 1771 | 1791 | UGGAGAAGAUACGCAAGAAAG | ACAUCUGGUGUGUCUCCUCUA |
| ath-miR838 | *MpARF6c* | 1 | 21 | 225 | 245 | UUUUCUUCUACUUCUUGCACA | GCCGCAGGAAGGAGAAAAAAG |
| ath-miR845b | *MpARF6c* | 1 | 22 | 46 | 67 | UCGCUCUGAUACCAAAUUGAUG | AGACGGUUUGGUGUUAGAUGGA |
| ath-miR172d-5p | *MpARF1b* | 1 | 21 | 1245 | 1265 | GCAACAUCUUCAAGAUUCAGA | GAUGAGAUUUGAAGGUGAUGA |
| ath-miR1888a | *MpARF1b* | 1 | 21 | 1961 | 1981 | UAAGUUAAGAUUUGUGAAGAA | AGCCUGAAAAAUCUUGCCUUA |
| ath-miR2934-3p | *MpARF1b* | 1 | 21 | 2348 | 2368 | CAUCCAAGGUGUUUGUAGAAA | CUGCACCAAGUACCUUGGAUG |
| ath-miR396a-5p | *MpARF1b* | 1 | 21 | 2047 | 2067 | UUCCACAGCUUUCUUGAACUG | GCUGUUGGGAGAGCUGUGGAU |
| ath-miR396b-5p | *MpARF1b* | 1 | 21 | 2047 | 2067 | UUCCACAGCUUUCUUGAACUU | GCUGUUGGGAGAGCUGUGGAU |
| ath-miR401 | *MpARF1b* | 1 | 21 | 1724 | 1744 | CGAAACUGGUGUCGACCGACA | AGUCUGUUGUCACUAGUGUCG |
| ath-miR8178 | *MpARF1b* | 1 | 21 | 1211 | 1231 | UAACAGAGUAAUUGUACAGUG | GGAAGCACAAUUUCUCUGUUG |
| ath-miR173-3p | *MpARF2c* | 1 | 21 | 2187 | 2207 | UGAUUCUCUGUGUAAGCGAAA | AAUUGUUUAUACGGAUAAUGA |
| ath-miR3932a | *MpARF2c* | 1 | 21 | 2082 | 2102 | AACUUUGUGAUGACAACGAAG | GUCUGUUGACCUUACAAAGUU |
| ath-miR3932b-3p | *MpARF2c* | 1 | 21 | 2082 | 2102 | AACUUUGUGAUGACAACGAAG | GUCUGUUGACCUUACAAAGUU |
| ath-miR417 | *MpARF2c* | 1 | 21 | 209 | 229 | GAAGGUAGUGAAUUUGUUCGA | GAGAGCUAGUUUUCUAUUUUC |
| ath-miR447c-3p | *MpARF2c* | 1 | 22 | 1037 | 1058 | UUGGGGACGACAUCUUUUGUUG | GCAUUGGAGAUGUUGAUCCCAA |
| ath-miR5632-5p | *MpARF2c* | 1 | 21 | 2127 | 2147 | UUGAUUCUCUUAUCCAACUGU | UGAGUUGGAUGAGAUGUUUGA |
| ath-miR5659 | *MpARF2c* | 1 | 23 | 2256 | 2278 | CGAUGAAGGUCUUUGGAACGGUA | CAUGGUUCGCAAGAUCUACAUCU |
| ath-miR779.2 | *MpARF2c* | 1 | 21 | 1458 | 1478 | UGAUUGGAAAUUUCGUUGACU | GUUCGGCUUAAUUUCUGGUCA |
| ath-miR838 | *MpARF2c* | 1 | 21 | 1507 | 1527 | UUUUCUUCUACUUCUUGCACA | CCUGCAGGAAGCAUGAGGGAA |
| ath-miR2938 | *MpARF19c* | 1 | 21 | 1161 | 1181 | GAUCUUUUGAGAGGGUUCCAG | UGAGAAUGCUUUCAAAAGGGC |
| ath-miR5020b | *MpARF3b* | 1 | 21 | 1667 | 1687 | AUGGCAUGAAAGAAGGUGAGA | UUUCACCAUCUUCUGUGCUAA |
| ath-miR5649a | *MpARF3b* | 1 | 21 | 1993 | 2013 | AUUGAAUAUGUUGGUUACUAU | UCAGUAGCGAAUGAAUUUAAU |
| ath-miR5649b | *MpARF3b* | 1 | 21 | 1993 | 2013 | AUUGAAUAUGUUGGUUACUAU | UCAGUAGCGAAUGAAUUUAAU |
| ath-miR869.2 | *MpARF3b* | 1 | 21 | 3 | 24 | UCUGGUGUUGAGAU-AGUUGAC | GGCAAUUGAUCUCAACACCAUC |
| ath-miR169a-5p | *MpARF9a* | 1 | 21 | 780 | 800 | CAGCCAAGGAUGACUUGCCGA | AAGUCAAUUCAUUGUUGGCUU |
| ath-miR169b-5p | *MpARF9a* | 1 | 21 | 780 | 800 | CAGCCAAGGAUGACUUGCCGG | AAGUCAAUUCAUUGUUGGCUU |
| ath-miR169c | *MpARF9a* | 1 | 21 | 780 | 800 | CAGCCAAGGAUGACUUGCCGG | AAGUCAAUUCAUUGUUGGCUU |
| ath-miR169d | *MpARF9a* | 1 | 21 | 781 | 801 | UGAGCCAAGGAUGACUUGCCG | AGUCAAUUCAUUGUUGGCUUG |
| ath-miR169e | *MpARF9a* | 1 | 21 | 781 | 801 | UGAGCCAAGGAUGACUUGCCG | AGUCAAUUCAUUGUUGGCUUG |
| ath-miR169f-5p | *MpARF9a* | 1 | 21 | 781 | 801 | UGAGCCAAGGAUGACUUGCCG | AGUCAAUUCAUUGUUGGCUUG |
| ath-miR169g-5p | *MpARF9a* | 1 | 21 | 781 | 801 | UGAGCCAAGGAUGACUUGCCG | AGUCAAUUCAUUGUUGGCUUG |
| ath-miR169h | *MpARF9a* | 1 | 21 | 780 | 800 | UAGCCAAGGAUGACUUGCCUG | AAGUCAAUUCAUUGUUGGCUU |
| ath-miR169i | *MpARF9a* | 1 | 21 | 780 | 800 | UAGCCAAGGAUGACUUGCCUG | AAGUCAAUUCAUUGUUGGCUU |
| ath-miR169j | *MpARF9a* | 1 | 21 | 780 | 800 | UAGCCAAGGAUGACUUGCCUG | AAGUCAAUUCAUUGUUGGCUU |
| ath-miR169k | *MpARF9a* | 1 | 21 | 780 | 800 | UAGCCAAGGAUGACUUGCCUG | AAGUCAAUUCAUUGUUGGCUU |
| ath-miR169l | *MpARF9a* | 1 | 21 | 780 | 800 | UAGCCAAGGAUGACUUGCCUG | AAGUCAAUUCAUUGUUGGCUU |
| ath-miR169m | *MpARF9a* | 1 | 21 | 780 | 800 | UAGCCAAGGAUGACUUGCCUG | AAGUCAAUUCAUUGUUGGCUU |
| ath-miR169n | *MpARF9a* | 1 | 21 | 780 | 800 | UAGCCAAGGAUGACUUGCCUG | AAGUCAAUUCAUUGUUGGCUU |
| ath-miR2934-5p | *MpARF9a* | 1 | 21 | 1683 | 1703 | UCUUUCUGCAAACGCCUUGGA | CUCAAAGCCUUUGAAGGAGGA |
| ath-miR3440b-3p | *MpARF9a* | 1 | 21 | 1364 | 1384 | UGGAUUGGUCAAGGGAAGCGU | AUGCUUCUCUUAACCUAUUCC |
| ath-miR414 | *MpARF9a* | 1 | 21 | 2007 | 2026 | UCAUCUUCAUCAUCAUCGUCA | UGAAGA-GGUGAAGAAGAUGA |
| ath-miR4221 | *MpARF9a* | 1 | 22 | 1685 | 1706 | UUUUCCUCUGUUGAAUUCUUGC | CAAAGCCUUUGAAGGAGGAAAA |
| ath-miR5648-3p | *MpARF9a* | 1 | 21 | 593 | 613 | AUCUGAAGAAAAUAGCGGCAU | AUGCUUUUGUUUUCUUAAGAG |
| ath-miR5650 | *MpARF9a* | 1 | 21 | 839 | 859 | UUGUUUUGGAUCUUAGAUACA | UUGGUAUGAGAUUCAAGAUGA |
| ath-miR390a-5p | *MpARF16b* | 1 | 21 | 931 | 951 | AAGCUCAGGAGGGAUAGCGCC | CGUGCUAGCACUCCCGAGUUU |
| ath-miR390b-5p | *MpARF16b* | 1 | 21 | 931 | 951 | AAGCUCAGGAGGGAUAGCGCC | CGUGCUAGCACUCCCGAGUUU |
| ath-miR396b-3p | *MpARF16b* | 1 | 21 | 610 | 630 | GCUCAAGAAAGCUGUGGGAAA | GAUUCAAUAGUGUUCUUGAGA |
| ath-miR5016 | *MpARF16b* | 1 | 21 | 1976 | 1996 | UUCUUGUGGAUUCCUUGGAAA | CAUUCAGUGAUUUCAUGAGAA |
| ath-miR834 | *MpARF16b* | 1 | 21 | 1567 | 1587 | UGGUAGCAGUAGCGGUGGUAA | ACACCGCGGCUAGUGCUAUUU |
| ath-miR843 | *MpARF16b* | 1 | 21 | 1767 | 1787 | UUUAGGUCGAGCUUCAUUGGA | ACCAAGUGAGCUCGGUUUGGA |
| ath-miR3440b-5p | *MpARF9b* | 1 | 21 | 118 | 138 | UUUUCUUGGCCCAUCCACUUC | UUGGUGGAUGUUCCAAGGAGG |
| ath-miR398a-3p | *MpARF9b* | 1 | 21 | 1327 | 1347 | UGUGUUCUCAGGUCACCCCUU | AUGGGGUGGCAUGAGGAUGUU |
| ath-miR398b-3p | *MpARF9b* | 1 | 21 | 1327 | 1347 | UGUGUUCUCAGGUCACCCCUG | AUGGGGUGGCAUGAGGAUGUU |
| ath-miR398c-3p | *MpARF9b* | 1 | 21 | 1327 | 1347 | UGUGUUCUCAGGUCACCCCUG | AUGGGGUGGCAUGAGGAUGUU |
| ath-miR406 | *MpARF9b* | 1 | 21 | 536 | 556 | UAGAAUGCUAUUGUAAUCCAG | AUGGUUUGGAGUGGCGUUUUA |
| ath-miR5647 | *MpARF9b* | 1 | 21 | 1520 | 1540 | UCAAGUUUGAUGACGAUUCCA | ACGAGUCCAUGUCAAGUUUGA |
